# Kinetic Properties Study of H Atom Abstraction by CH_3_Ȯ_2_ Radicals from Fuel Molecules with Different Functional Groups

**DOI:** 10.1021/acs.jpca.2c08100

**Published:** 2023-02-20

**Authors:** Hao-Ting Guo, Yan Tang, Sheng-Han Liu, Yang Ma, Shen Fang, Henry J. Curran, Chong-Wen Zhou

**Affiliations:** † School of Energy and Power Engineering, Beihang University, Beijing 100191, China; ‡ Combustion Chemistry Centre, School of Biological and Chemical Sciences, Ryan Institute, University of Galway, Galway H91TK33, Ireland

## Abstract

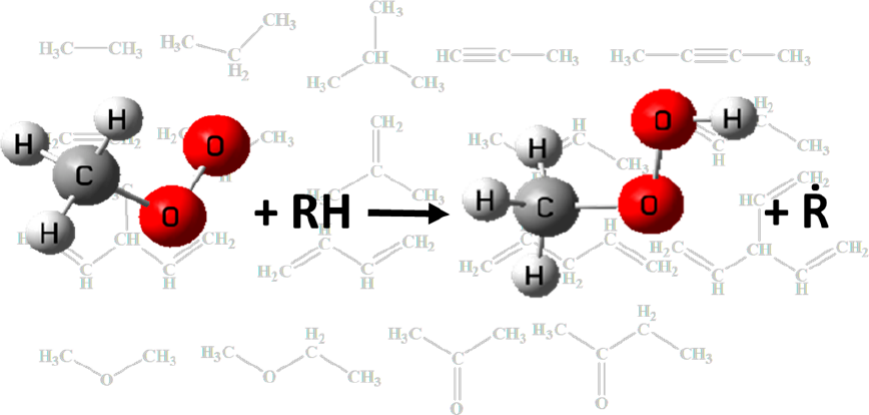

The detailed kinetic properties of hydrogen atom abstraction by methylperoxy (CH_3_Ȯ_2_) radicals from alkanes, alkenes, dienes, alkynes, ethers, and ketones are systematically studied in this work. Geometry optimization, frequency analysis, and zero-point energy corrections were performed for all species at the M06-2X/6-311++G(d,p) level of theory. The intrinsic reaction coordinate calculation was consistently performed to ensure that the transition state connects the correct reactants and products, and one-dimensional hindered rotor scanning results were performed at the M06-2X/6-31G level of theory. The single-point energies of all reactants, transition states, and products were obtained at the QCISD(T)/CBS level of theory. High-pressure-limit rate constants of 61 reaction channels were calculated using conventional transition state theory with asymmetric Eckart tunneling corrections over the temperature range of 298.15–2000 K. Reaction rate rules for H atom abstraction by CH_3_Ȯ_2_ radicals from fuel molecules with different functional groups are constructed, which can be used in the development of combustion models of these fuels and fuel types. In addition, the influence of the functional groups on the internal rotation of the hindered rotor is also discussed.

## 1 Introduction

The utilization of energy is closely related to advancements in the quality of life of human society. At present, 83% of the world’s energy is provided by fossil fuels. From 2005 to 2020 the global primary energy consumption structure has undergone major changes, and the proportion of coal and oil consumption has shown a downward trend. The COVID-19 pandemic that swept the world in 2020 led to a recession in the global economy. The world’s total primary energy consumption fell by 4.5%, but the proportion of oil consumption still topped the list at 31.2%, with coal at 27.2% and natural gas at 24.7%. Although the consumption of fossil energy has decreased, it is still the dominant energy source, and this is set to persist into the future. As an important part of fossil energy, alkane fuels are widely used in civil and industrial applications. Alkene fuels are an important part of commercial fuels and are important intermediates in the oxidation of hydrocarbon species.^1^ The presence of double bonds in alkene molecules increases the complexity of the chemical reactions involved in fuel combustion, and compared to alkanes, alkenes can generate more radical species during reaction.^2−4^ Alkynes such as acetylene, which contain carbon–carbon triple bonds, are easily polymerized to form pollutants such as aromatics and are important precursors of soot formation.^5−7^

Meanwhile, increasing attention to the ecological environment has promoted research into new energy sources, including oxygenated biofuels. Ethers are widely used in experimental research and industrial applications due to their good solvency and chemical inertness, and ethers can also be used as additives in unleaded gasoline to increase the octane number.^8,9^ In addition, they can be applied as an alternative fuel for reformulated gasoline, which can contribute to reductions in CO and NO_*x*_ emissions without compromising performance.^10^ Ketones are often used as industrial coatings and solvents^11^ and are pollutants. In atmospheric and combustion systems they are intermediates in the oxidation of hydrocarbon species during combustion. In addition, because of their fluorescent properties, they are used as fuel tracers to make non-invasive measurements of temperature fields and reactant composition in difficult-to-handle environments such as internal combustion engines.^12^ Understanding the reaction properties of ketones, especially at temperatures above 500 K, is important to the development of detailed chemical kinetic models applicable to combustion systems.

Hydrogen atom abstraction reactions by methylperoxy radicals (CH_3_Ȯ_2_) are particularly important in fuel combustion. Curran^13^ noted that CH_3_Ȯ_2_ is an important hydrogen abstractor at intermediate to high temperatures (800–1250 K), particularly for fuels that produce relatively high concentrations of methyl radicals (ĊH_3_). According to the experimental results of Fernandes et al.,^14^ the rate constant for the formation of CH_3_Ȯ_2_ from methyl radicals and oxygen increases with increasing pressure in the temperature and pressure ranges of 300–700 K and 2–1000 bar.

According to Pilling,^15^ alkylperoxyl radicals, ROȮ, can produce alkyl hydroperoxide (ROOH) species via hydrogen atom abstraction reactions (ROȮ + RH → ROOH + R̈), and ROOH can subsequently decompose to generate alkoxy and ȮH radicals at *T* > 850 K. As one of the ROȮ radicals, CH_3_Ȯ_2_ can generate CH_3_OOH via hydrogen atom abstraction reactions, and CH_3_OOH can rapidly decompose to CH_3_Ȯ and ȮH radicals, which are highly reactive and can greatly increase fuel reactivity. In a modeling study of ethanol/gasoline blending, Cheng et al.^16^ found that hydrogen abstraction reactions from fuels by methylperoxy radicals are important in the temperature range 700–1000 K. According to the comprehensive mechanism developed for iso-octane oxidation by Curran et al.^17^ at high pressures the CH_3_Ȯ_2_ radicals stabilize, and hydrogen atom abstraction reactions by CH_3_Ȯ_2_ are important at 1000 K.^17^ In the study of CH_4_/DME blends by Burke et al.,^18^ hydrogen atom abstraction by CH_3_Ȯ_2_ radicals was found to be important in the temperature range 706–1250 K. Carstensen et al.^19,20^ calculated the rate constants for the abstraction of hydrogen atoms by CH_3_Ȯ_2_ radicals from ethane, propane, and iso-butane at the CBS-QB3//B3LYP/CBSB7 level of theory. Yang et al.^21^ calculated hydrogen atom abstraction from the components of the FGF-LLNL gasoline surrogate, including cyclopentane, toluene, 1-hexene, *n*-heptane, and iso-octane, by CH_3_Ȯ_2_ radicals at the QCISD(T)/CBS//M06-2X/6-311++g(d,p) level of theroy. However, abstraction of hydrogen atom by CH_3_Ȯ_2_ radicals from species containing different functional groups, such as carbon–carbon double bonds (C=C), carbon–carbon triple bonds (C≡C), ethers (−O−), and carbonyl groups (−C=O), have not been studied. Thus, a systematic study of the influence of functional groups will be helpful in understanding the combustion of different types of fuels. With this in mind, a detailed theoretical study of the rate constants for H atom abstraction by CH_3_Ȯ_2_ radicals from alkanes, alkenes, dienes, alkynes, ethers, and ketones is performed. The individual rate constants for a series of abstractions from different functional groups are provided, and the effect of the functional groups on the rate constants for abstraction is also investigated.

## 2 Computational Methods

### 2.1 Potential Energy Surfaces

In this study, the M06-2X^22^ method combined with the 6-311++G(d,p)^23,24^ basis set is used to perform the geometry optimizations, vibrational frequencies, and zero-point energy (ZPE) calculations for all reactants, transition states, and products. The intrinsic reaction coordinate^25^ calculations are also performed at the same level of theory to ensure that the reactants and products are correctly connected by the transition state. The low-frequency torsional modes associated with C–C, C–O, and O–O single bonds of all transition states, reactants, and products are treated with a one-dimensional hindered rotor at the M06-2X/6-31G^26^ level of theory. The single-point energies (SPEs) of the individual species are obtained using the QCISD(T)^27^ method by combining the cc-pVDZ^26^ and cc-pVTZ^28^ basis sets and the second-order Møller–Plesset theory (MP2)^29^ correction with cc-pVDZ, cc-pVTZ, and cc-pVQZ^30^ basis sets. The SPEs are extrapolated to the complete basis set (CBS) limit using the following equation:^31^
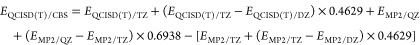
1A correction factor of 0.9698^22^ is used to correct the ZPEs. The T1 diagnostics^32^ for all of the species investigated in this study are less than 0.035, which indicates that the single-reference method can reliably obtain the electronic energy for the species in this study. Geometry optimization, frequency, and IRC calculations are carried out using the Gaussian 16^33^ program, and the SPE calculations are carried out using Molpro.^34^

### 2.2 Rate Constant Calculations

Conventional transition state theory is used to obtain the high-pressure-limit rate constants for reaction channels involved in the temperature range of 298.15–2000 K:
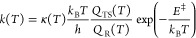
where *k*_B_ is Boltzmann’s constant, *h* is Planck’s constant, and *E*^⧧^ is the energy barrier height. κ(*T*) is the transmission coefficient, which accounts for tunneling effects using the one-dimensional asymmetric Eckart model.^35^*Q*_R_ and *Q*_TS_ are the partition functions of the reactants and the transition state, respectively. The partition function of each molecule, radical, or transition state is obtained by calculating and multiplying the translational (*Q*_trans_), vibrational (*Q*_vib_), rotational (*Q*_rot_), electronic (*Q*_el_), and torsional (*Q*_tor_) partition functions. Due to the complexity of the processing of vibration patterns by the vibrational partition function,^36^ we use the ProjRot^37^ program to obtain the vibrational frequencies without torsional modes from the Hessian matrix calculated at the M06-2X/6-311++G(d,p) level of theory. All of the rate constants are calculated using the Master Equation System Solver (MESS)^38^ program. The rate constant results are fitted to the modified Arrhenius expression *k* = *AT*^*n*^ exp(−*E*_a_/*RT*), and the fitted values of *A*, *n*, and *E*_a_ are provided in the Supporting Information.

## 3 Results and Discussion

### 3.1 Calculation of Species and Reaction Sites

**Figure 1:**
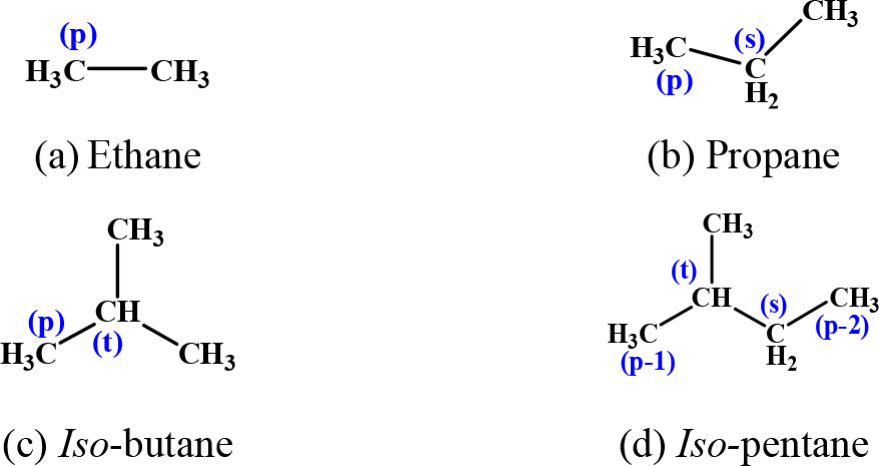
Alkanes investigated in this work.

**Figure 2:**
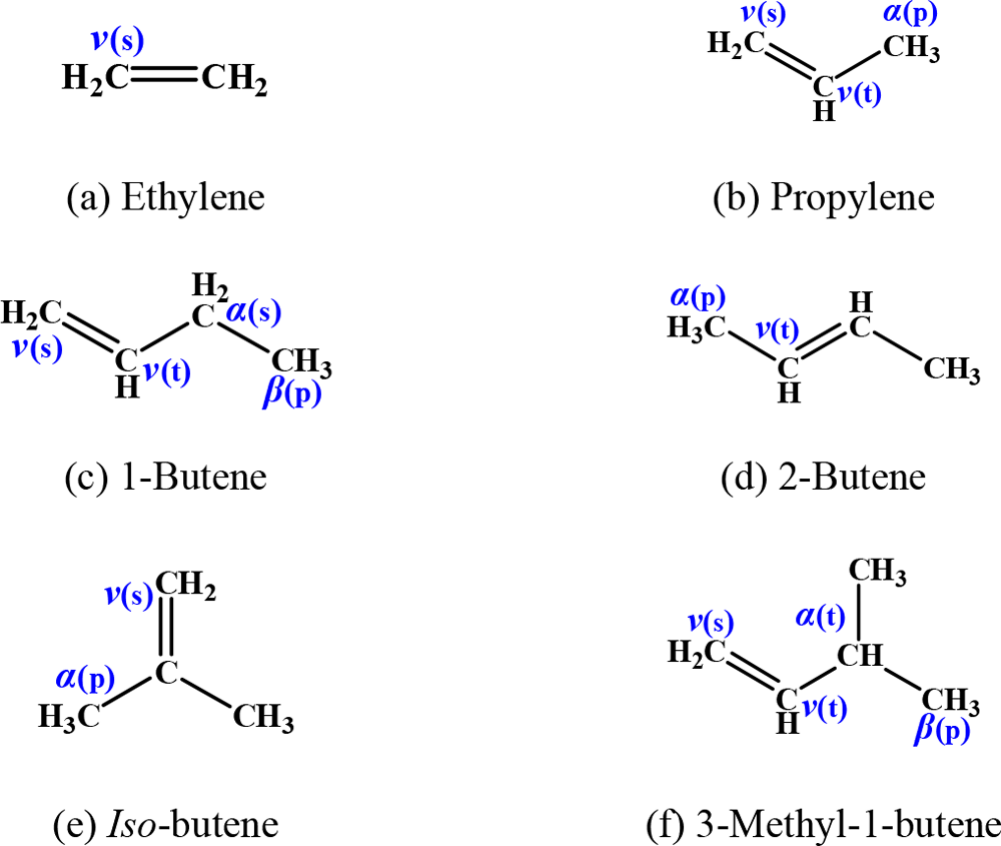
Alkenes investigated in this work.

**Figure 3:**
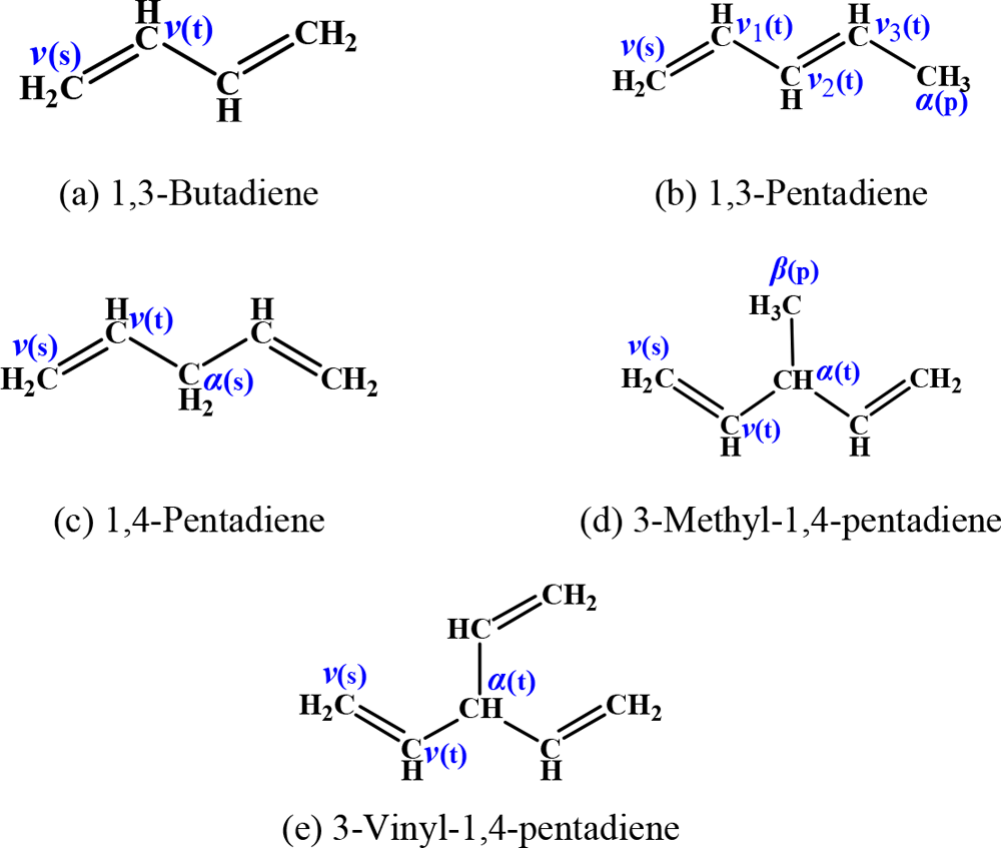
Dienes and a triene investigated in this work.

**Figure 4:**
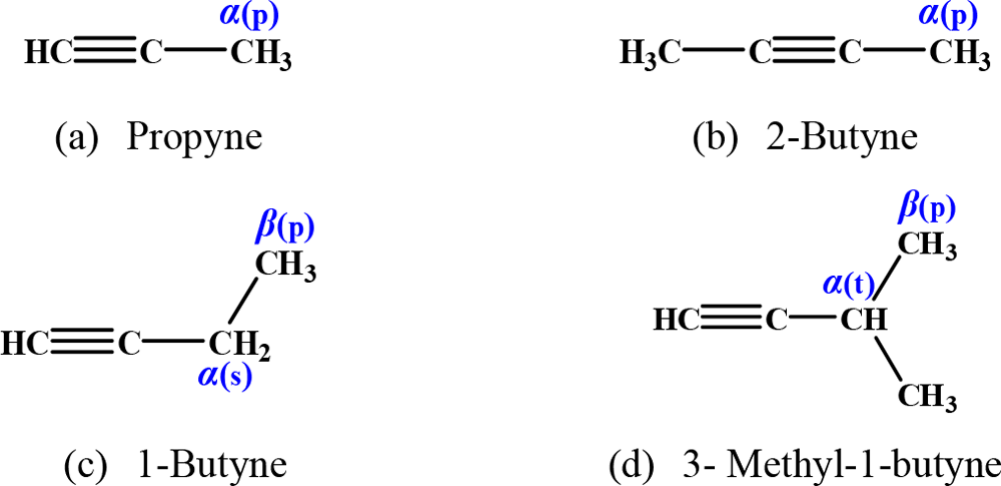
Alkynes investigated in this work.

**Figure 5:**
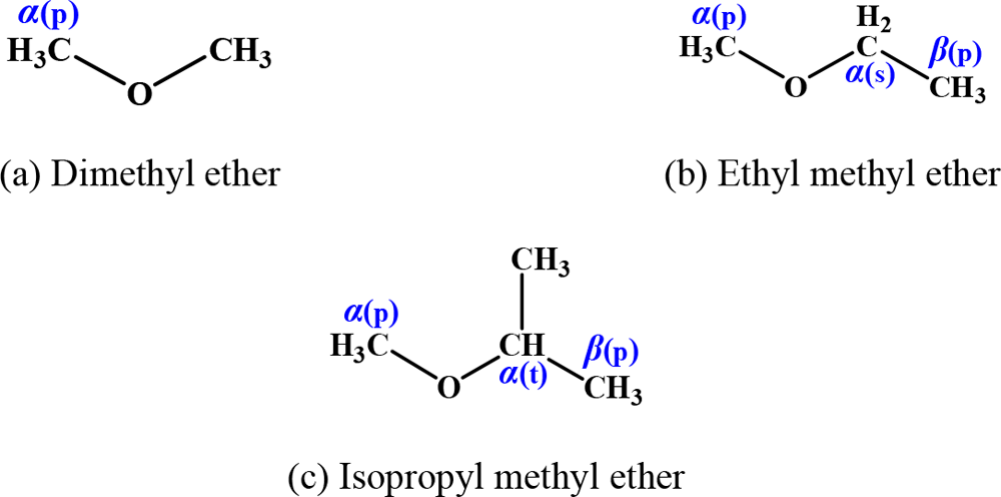
Ethers investigated in this work.

**Figure 6:**
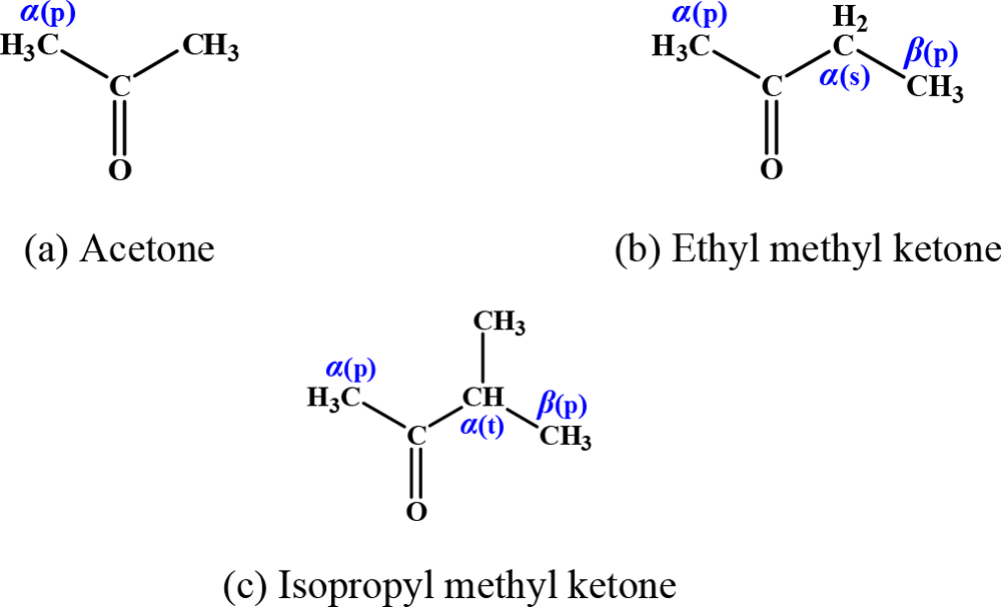
Ketones investigated in this work.

A large number of different functional group species are involved in this study, with a total of 26 reactants and 61 reaction channels. The reactant species and sites for alkanes, alkenes, dienes, alkynes, ethers, and ketones are summarized in Figures 1–6. To illustrate in detail all of the different types of hydrogen atoms studied, we have grouped all of the calculated sites according to the type of carbon on which the hydrogen atom is located. For the alkanes in Figure 1, the three different types of carbon atoms are primary (labeled p), secondary (labeled s), and tertiary (labeled t). For species containing functional groups, the carbon atoms are labeled as α and β considering their distance from the functional group. In particular, we define the carbon connecting the double bond in the alkenes to the vinylic carbon as a *v* carbon atom.

### 3.2 Electronic Energy Barriers

Tables 1–6 present the calculated electronic energy barriers for H atom abstraction by CH_3_Ȯ_2_ radicals from alkanes, alkenes, dienes, alkynes, ethers, and ketones, respectively. Table 1 shows the energy barriers for H atom abstraction from the four target alkanes at different sites. The highest average energy barrier is 19.83 kcal mol^–1^ for H atom abstraction at a primary (1°) carbon site, decreasing to 17.01 kcal mol^–1^ for abstraction from a secondary (2°) carbon site and 14.76 kcal mol^–1^ for abstraction from a tertiary (3°) carbon site. This stepwise decrease in activation energy is consistent with the bond dissociation energies of the different (1°, 2°, and 3°) C–H bonds.

**Table 1:** Comparison of the Energy Barriers (in kcal mol^–1^) at the QCISD(T)/CBS Level of Theory for H Atom Abstraction by CH_3_Ȯ_2_ Radicals from Different Sites (Primary, Secondary, or Tertiary) on Alkane Species

species	primary	secondary	tertiary
ethane	20.19	–	–
propane	19.47	17.48	–
isobutane	19.60	–	15.02
isopentane	20.44,^a^ 19.42^b^	16.54	14.50
average	19.83	17.01	14.76

a Energy barrier for the carbon labeled as p-1 in Figure 1d.

b Energy barrier for the carbon labeled as p-2 in Figure 1d.

Table 2 summarizes the energy barriers for H atom abstraction from the six target alkenes at different sites. Due to the presence of the double bonds in alkenes, the carbon atoms can be classified into allylic, vinylic, and alkylic carbon atoms based on their proximity to the double bond. The data indicate that the energy barriers for abstraction from allylic carbon atoms are significantly lower than those from alkylic carbon atoms, whereas abstractions from vinylic carbon atoms have the highest energy barriers. This is due to the fact that, after the hydrogen is abstracted from the allylic site, the carbon atoms on both sides of the carbon–carbon double bond become sp^2^-hybridized, thus forming a resonance-stabilized radical,^39^ making these hydrogen atoms more easily abstracted. The lowest energy barrier is 12.28 kcal mol^–1^ for H atom abstraction from a tertiary allylic carbon atom, and the highest energy barrier is 24.34 kcal mol^–1^ for abstraction of a hydrogen atom from a secondary vinylic carbon atom.

**Table 2:** Comparison of the Energy Barriers (in kcal mol^–1^) at the QCISD(T)/CBS Level of Theory for H Atom Abstraction by CH_3_Ȯ_2_ Radicals from Different Sites on Monoalkenes

	allylic (α)			vinylic (*v*)		alkylic (β)
species	primary	secondary	tertiary	secondary	tertiary	primary
ethylene	–	–	–	24.55	–	–
propylene	16.17	–	–	24.48	21.53	–
1-butene	–	13.34	–	24.49	21.16	20.29
2-butene	14.82	–	–	–	21.58	–
isobutene	15.24	–	–	24.39	–	–
3-methyl-1-butene	–	–	12.28	23.79	21.19	20.64
average	15.41	13.34	12.28	24.34	21.37	20.47

The energy barriers for abstraction from the four target dienes and the one target triene at different sites are listed in Table 3. The energy barrier for abstraction of hydrogen atoms at the allylic site is significantly lower than that from the alkylic site, while abstraction from the vinylic site has the highest energy barrier. For the vinylic sites, the energy barriers for abstraction from 3-methyl-1,4-pentadiene and 3-vinyl-1,4-pentadiene are approximately 1.0 kcal mol^–1^ lower compared to those from 1,3-butadiene, 1,3-pentadiene, and 1,4-pentadiene, which may be related to further electron delocalization due to the additional methyl groups.

**Table 3:** Comparison of the Energy Barriers (in kcal mol^–1^) at the QCISD(T)/CBS Level of Theory for H Atom Abstraction by CH_3_Ȯ_2_ Radicals from Different Sites on Diene and Triene Species

	allylic (α)			vinylic (*v*)		alkylic (β)
species	primary	secondary	tertiary	secondary	tertiary	primary
1,3-butadiene	–	–	–	24.98	22.20	–
1,3-pentadiene	13.37	–	–	24.72	21.63,^a^ 22.24,^b^ 21.91^c^	–
1,4-pentadiene	–	11.47	–	24.29	21.77	–
3-methyl-1,4-pentadiene	–	–	9.26	23.63	20.70	19.36
3-vinyl-1,4-pentadiene	–	–	8.54	23.46	20.83	–
average^d^	13.37	11.47	9.26	24.41	21.74	19.36

a Energy barrier for the carbon labeled as *v*_1_(t) in Figure 3b.

b Energy barrier for the carbon labeled as *v*_2_(t) in Figure 3b.

c Energy barrier for the carbon labeled as *v*_3_(t) in Figure 3b.

d Average: The average energy barrier of H atom abstraction from four target dienes at the corresponding positions.

The energy barriers for abstraction from the four target alkynes are shown in Table 4. It can be seen that the energy barrier for abstraction from the propargylic carbon site, which is close to the carbon–carbon triple bond, is significantly lower than that from the alkylic carbon site. The lowest energy barrier is 12.7 kcal mol^–1^ for abstraction from a tertiary propargylic carbon atom, and the highest energy barrier is 19.67 kcal mol^–1^ for abstraction from a primary alkylic carbon site.

**Table 4:** Comparison of the Energy Barriers (in kcal mol^–1^) at the QCISD(T)/CBS Level of Theory for H Atom Abstraction by CH_3_Ȯ_2_ Radicals from Different Sites on Alkyne Species

	propargylic (α)			alkylic (β)
species	primary	secondary	tertiary	primary
propyne	16.62	–	–	–
2-butyne	15.27	–	–	–
1-butyne	–	13.95	–	19.67
3-methyl-1-butyne	–	–	12.70	–
average	15.95	13.95	12.70	19.67

Table 5 lists the energy barriers for abstraction from three ethers with different sites. The energy barrier for abstraction from an α carbon atom adjacent to the −O– atom in ethers is significantly lower than that for abstraction from a β carbon atom. According to valence bond theory, the bonds at the α site adjacent to the ether functional group are stabilized by the oxygen lone pair, which lowers the bond energy at these sites. This conjugation effect significantly reduces the energy barrier for abstraction, with the lowest energy barrier being 12.45 kcal mol^–1^ for abstraction from an α-tertiary carbon atom.

**Table 5:** Comparison of the Energy Barriers (in kcal mol^–1^) at the QCISD(T)/CBS Level of Theory for H Atom Abstraction by CH_3_Ȯ_2_ Radicals from Different Sites on Ethers

	adjacent (α) to the ether functional group			alkylic (β)
species	primary	secondary	tertiary	primary
dimethyl ether	15.52	–	–	–
ethyl methyl ether	16.18	14.16	–	21.52
isopropyl methyl ether	14.23	–	12.45	20.56
average	15.31	14.16	12.45	21.04

Table 6 lists the energy barriers for H atom abstraction from three ketones at different sites. Similar to ethers, the energy barriers for H atom abstraction at the position α to the functional group are significantly lower than those for alkylic carbon sites. In particular, the energy barrier for abstraction from an α-tertiary carbon atom has the lowest energy barrier of 13.38 kcal mol^–1^.

**Table 6:** Comparison of the Energy Barriers (in kcal mol^–1^) at the QCISD(T)/CBS Level of Theory for H Atom Abstraction by CH_3_Ȯ_2_ Radicals from Different Sites on Ketones

	adjacent to the carbonyl functional group (α)			alkylic (β)
species	primary	secondary	tertiary	primary
acetone	17.38	–	–	–
ethyl methyl ketone	17.13	15.22	–	18.92
isopropyl methyl ketone	16.98	–	13.38	19.63
average	17.16	15.22	13.38	19.28

**Figure 7:**
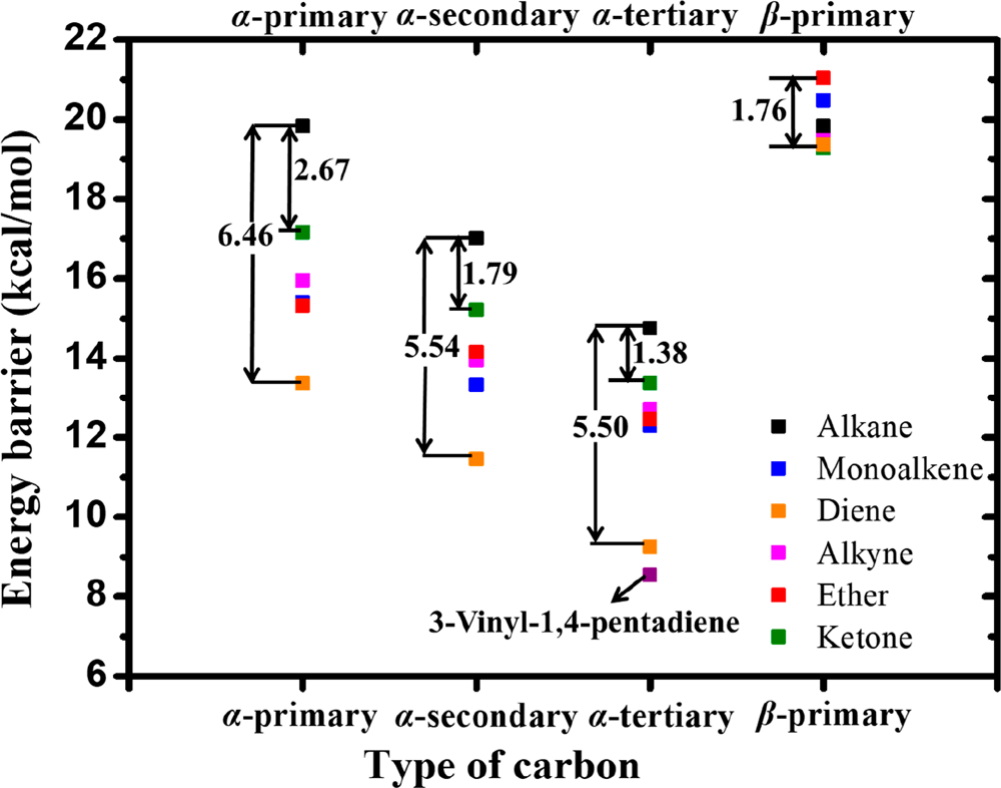
Comparison of the average energy barriers for H atom abstraction by CH_3_Ȯ_2_ from alkanes (black), monoalkenes (blue), dienes (orange), the triene 3-vinyl-1,4-pentadiene (purple), alkynes (magenta), ethers (red), and ketones (green) at the α-primary, α-secondary, α-tertiary, and β-primary carbon sites for different functional groups.

The average energy barriers for abstraction at similar sites from species with the five different functional groups are compared with those for alkanes (without functional groups), and the results are summarized in Figure 7 and Table 7. The overall trend shows that the α-tertiary carbon site adjacent to the functional group has the lowest energy barrier, followed by secondary and primary carbon atoms, with the β sites having the highest energy barriers.

**Table 7:** Comparison of Average Energy Barriers (in kcal mol^–1^) at the QCISD(T)/CBS Level of Theory for H-Atom Abstraction at Similar Locations from Species with Different Functional Groups

	adjacent to the functional group (α)			alkylic (β)
species	primary	secondary	tertiary	primary
alkane	19.83	17.01	14.76	–
monoalkene	15.41	13.34	12.28	20.47
diene	13.37	11.47	9.26	19.36
triene	–	–	8.96	–
alkyne	15.95	13.95	12.7	19.67
ether	15.31	14.16	12.45	21.04
ketone	17.16	15.22	13.38	19.28

The presence of dienes has the strongest effect on the energy barrier for H atom abstraction by CH_3_Ȯ_2_ radicals, showing the largest decrease compared to alkanes, whereas ketones show the smallest decrease. For the α-primary carbon atom, the diene leads to an energy barrier for abstraction of 13.37 kcal mol^–1^, which is 6.46 kcal mol^–1^ lower than that for abstraction from a primary carbon site in an alkane. The carbonyl functional group in ketones leads to an energy barrier of 17.16 kcal mol^–1^, which is 2.67 kcal mol^–1^ lower compared to the equivalent alkane. For α-secondary carbon atoms in dienes, the energy barrier for abstraction is 11.47 kcal mol^–1^, which is 5.54 kcal mol^–1^ lower than that for the corresponding secondary alkane carbon site, while that for ketones is only 1.79 kcal mol^–1^ lower compared to the alkane. The energy barrier of 9.26 kcal mol^–1^ for α-tertiary carbon atom abstraction from a diene is 5.50 kcal mol^–1^ lower than that from the comparable site in an alkane, while the barrier for abstraction from a ketone is only 1.38 kcal mol^–1^ lower than that of the alkane. Finally, we calculated an energy barrier of 8.54 kcal mol^–1^ for abstraction from an α-tertiary carbon atom in 3-vinyl-1,4-pentadiene, thus indicating that a triene has an even lower energy barrier than a diene.

Based on these results, we conclude that the presence of functional groups has the strongest effect on the energy barrier for abstraction at the α-primary carbon site, showing the largest reduction in energy barrier compared to alkanes. This is followed by abstraction from the secondary and tertiary carbon sites, respectively. The β carbon sites show a much lower influence on the barrier height due to their greater distance from the functional group, with the barrier heights being similar to those of the corresponding alkanes. Table 7 gives the average energy barriers for H atom abstraction from fuels with different functional groups at similar sites.

### 3.3 Rate Constants and Rate Rules

**Figure 8:**
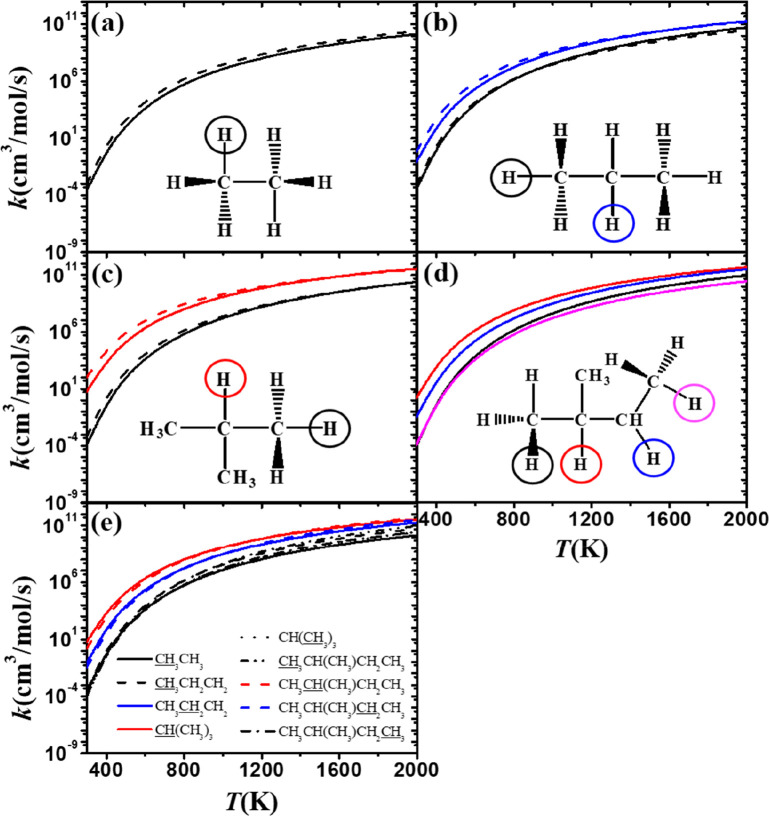
(a–d) Comparison of rate constants for H atom abstraction (on a per hydrogen atom basis) by CH_3_Ȯ_2_ radicals at different sites on (a) ethane, (b) propane, (c) isobutane, and (d) isopentane. The solid lines show calculations from this work at the QCISD(T)/MP2/CBS//M06-2X/6-311++G(d,p) level of theory, and the dashed lines in (a–c) show data from Carstensen et al.^20^ at the CBS-QB3//B3LYP/CBSB7 level of theory. (e) Summary of rate constants from all sites.

**Figure 9:**
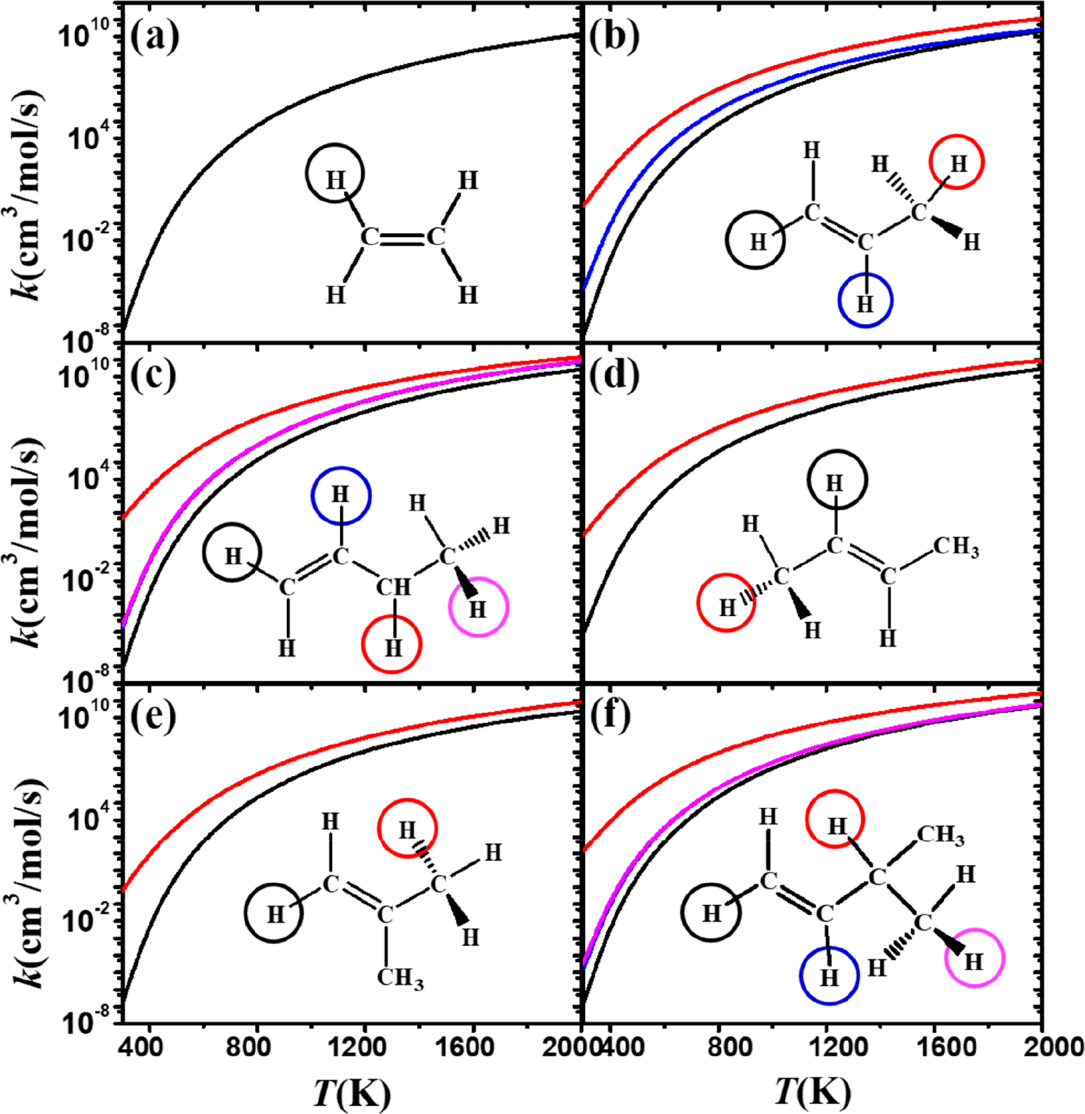
Comparison of rate constants, on a per H atom basis, for abstraction by CH_3_Ȯ_2_ radicals at different sites on (a) ethylene, (b) propylene, (c) 1-butene, (d) 2-butene, (e) isobutene, and (f) 3-methyl-1-butene.

**Figure 10:**
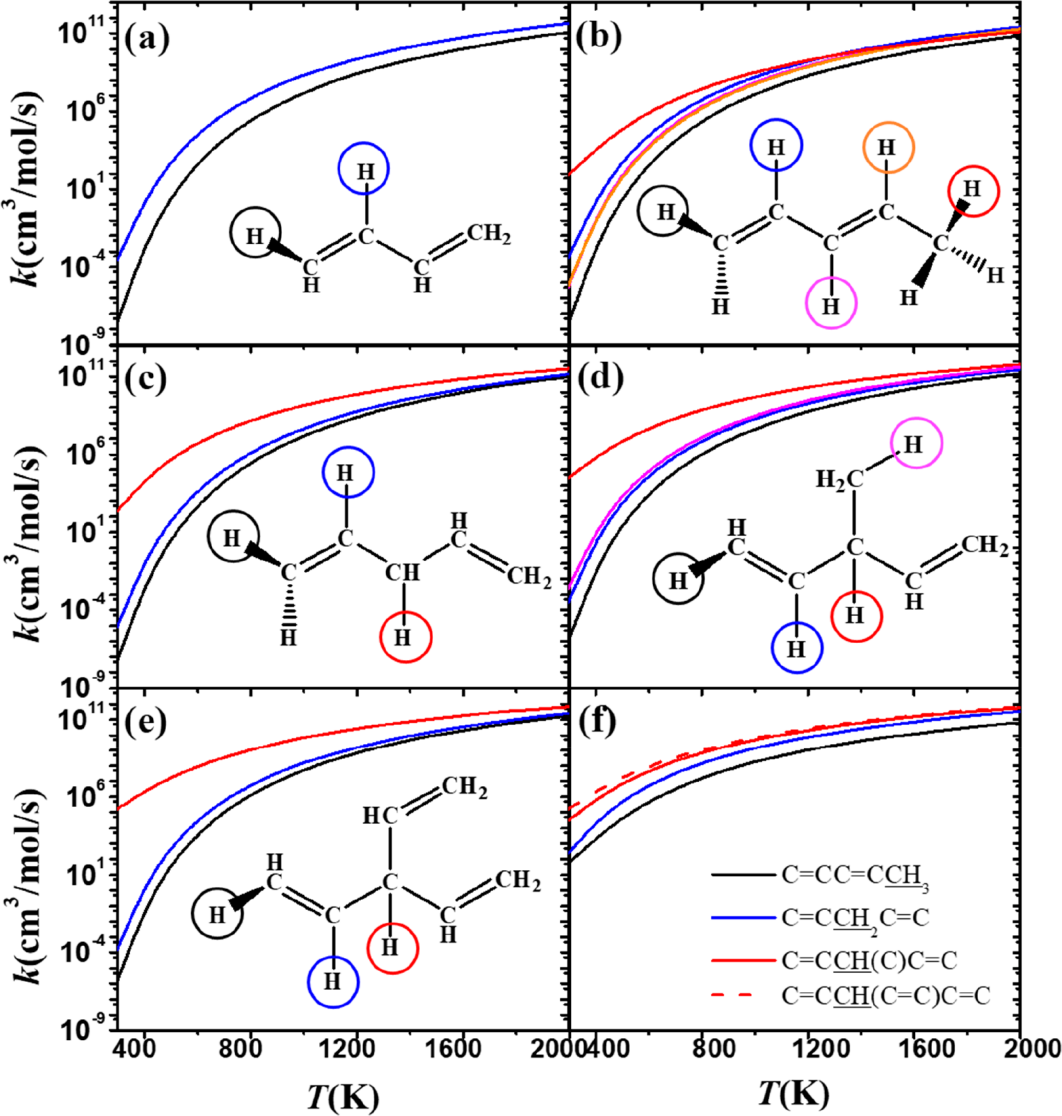
Comparison of rate constants, on a per hydrogen atom basis, for abstraction by CH_3_Ȯ_2_ radicals at different sites on (a) 1,3-butadiene, (b) 1,3-pentadiene, (c) 1,4-pentadiene, (d) 3-methyl-1,4-pentadiene, and (e) 3-vinyl-1,4-pentadiene. (f) Summary of rate constants for all α sites in (b–e).

**Figure 11:**
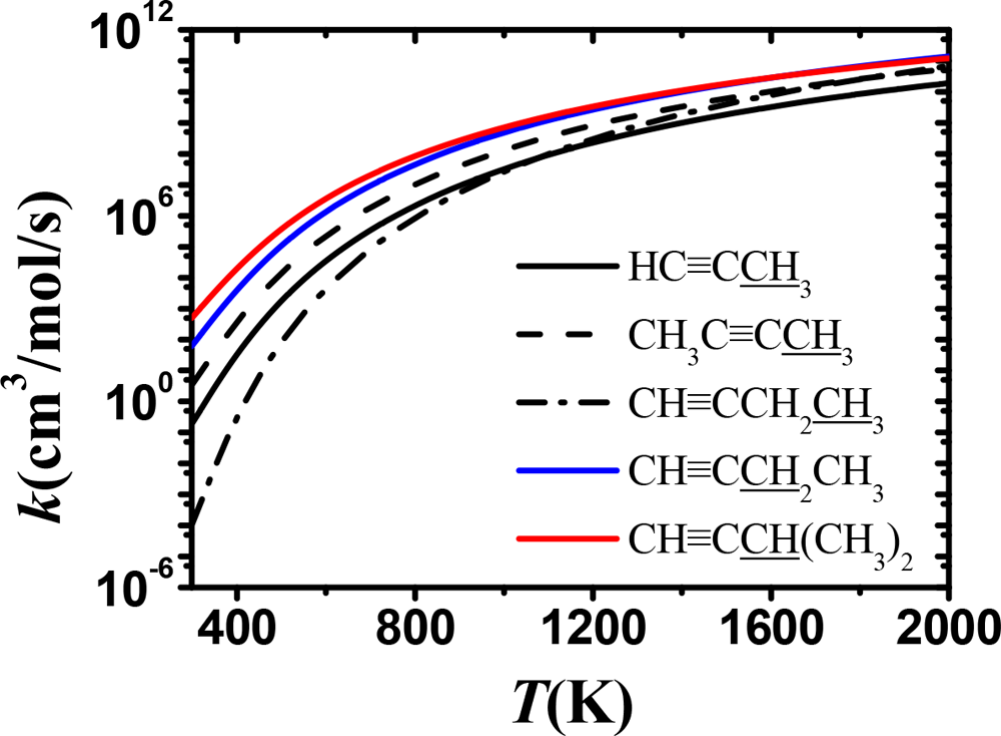
Comparison of rate constants, on a per hydrogen atom basis, for H atom abstraction by CH_3_Ȯ_2_ radicals from propyne (black solid line), 2-butyne (dashed line), 1-butyne (dotted line), and 3-methyl-1-butyne. The blue line is the secondary carbon site and the red line is the tertiary carbon site on 1-butyne.

**Figure 12:**
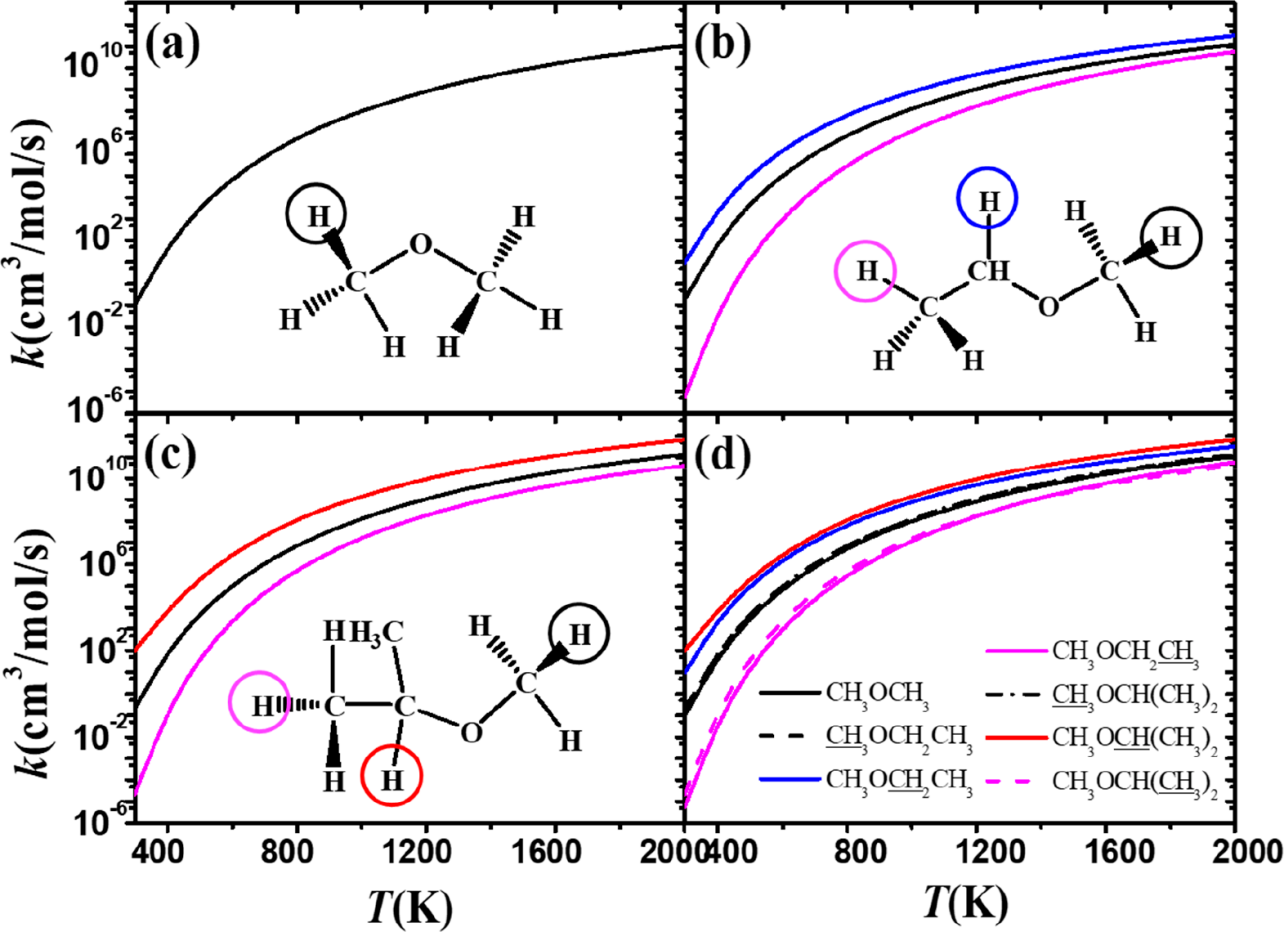
Comparison of rate constants, on a per H atom basis, for abstraction by CH_3_Ȯ_2_ radicals at different sites on (a) dimethyl ether, (b) methyl ethyl ether, and (c) methyl isopropyl ether. (d) Summary of rate constants from all sites.

**Figure 13:**
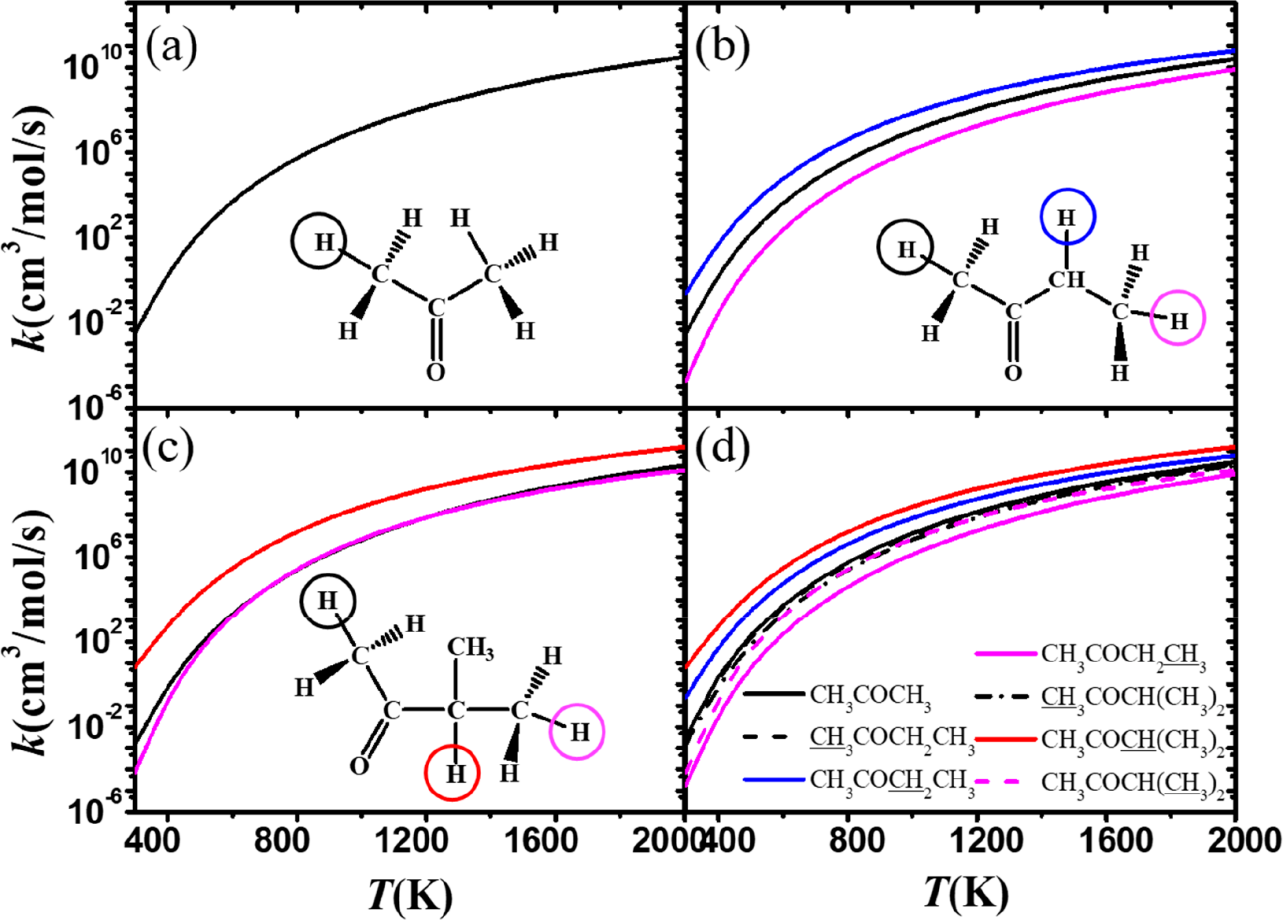
Comparison of rate constants, on a per hydrogen atom basis, for abstraction by CH_3_Ȯ_2_ radicals at different sites on (a) acetone, (b) methyl ethyl ketone, and (c) methyl isopropyl ketone. (d) Summary of rate constants from all sites.

The calculated high-pressure-limit rate constants in the temperature range of 298.15–2000 K for abstraction from species with different functional groups, including alkanes, alkenes, dienes, alkynes, ethers, and ketones, are shown in Figures 8–13. The effect of abstraction from the same species at different sites and the effect of abstraction from different functional groups the same site are discussed separately.

#### 3.3.1 High-Pressure-Limit Rate Constants and Branching Ratios

Figure 8 shows a comparison of rate constants on a per hydrogen atom basis for abstraction from different sites on alkanes. The general trend for all alkanes is that the rate constant for abstraction at the tertiary carbon site is higher than that from the secondary site, which is higher than that from the primary carbon site (Figure 8e).

In the case of iso-pentane (Figure 8d), for example, at 500 K the rate constant for abstraction from the tertiary carbon site (red lines) is about 183 times higher than that at the primary carbon site (black line). Moreover, the rate constant for abstraction from the secondary carbon site (blue lines) is about 37 times higher than that from the primary carbon site (black lines). The difference in rate constants decreases with increasing temperature. At 2000 K the rate constant for abstraction from the tertiary carbon site is 5.2 times higher compared to the primary carbon site, and that for abstraction from the secondary carbon site is 3.6 times higher than that from the primary carbon site. In addition, the present work compares our rate constants (solid lines) for abstraction by CH_3_Ȯ_2_ radicals from ethane, propane, and iso-butane with those published previously by Carstensen et al.^20^ (dashed lines). The comparisons in Figure 8a–c show that the rate constants calculated by Carstensen et al. are slightly higher than our calculations, mainly from the different computational methods used. In their calculations, the results were obtained at the CBS-QB3//B3LYP/CBSB7 level of theory.^20^

The rate constants for abstraction reactions (on a per hydrogen atom basis) from monoalkenes are reported in Figure 9. The rate constants for abstraction from the α carbon sites (red lines) are highest, with the lowest being those for abstraction from the vinylic site (black lines). At low temperatures, the rate constant for abstraction from the α site is 7–8 orders of magnitude higher comparted to that for abstraction from the vinylic site. It is noteworthy that for 3-methyl-1-butene (Figure 9f) the rate constant is highest at the α(t) carbon site (red line) and lowest for abstraction from a *v*(s) carbon site (black line). However, the rate constants of abstraction from tertiary carbon sites (blue lines) tend to be the same as from β(p) sites (magenta lines), which is related to the similar energy barriers calculated for abstraction from both sites.

Figure 10 shows rate constant comparisons for abstraction from different sites on four dienes and one triene. Similar to monoalkenes discussed above, abstraction from the α carbon site (red lines) is the fastest, followed by the *v*(t) carbon site (blue lines), with abstraction from the *v*(s) carbon site being the slowest (black lines). Figure 10f summarizes the rate constants for all α sites in the three dienes and one triene shown in Figure 10b–e. The rate constant for abstraction from the α(t) carbon site on 3-vinyl-1,4-pentadiene (red dashed line in Figure 10f) is slightly higher than that for 3-methyl-1,4-pentadiene (red solid line). This is followed by abstraction from the α(s) site on 1,4-pentadiene and finally from the α(p) site on 1,3-pentadiene. The difference in the rate constants for abstraction from the α(t) and α(p) sites is a factor of about 400 at 500 K. At high temperatures (*T* > 1800 K), the difference decreases to an order of magnitude.

We have also performed a comparison of calculated rate constants for abstraction from different sites in alkynes, ethers, and ketones, which are presented in Figures 11–13. Rate constants for abstraction from five different sites were calculated for the alkynes. Figure 11 shows that the highest rate constants are for abstraction from the α(t) carbon site (red line) on 3-methyl-1-butyne, followed by the α(s) carbon site of 1-butyne (blue line). The black line indicates the primary carbon site, and the rate constant of hydrogen abstraction from the β carbon site on 1-butyne (dash-dotted line), which is further away from the C≡C triple bond, is much lower than that for abstraction from the α(p) site at low temperatures. Moreover, the rate constant for abstraction from the β site increases more rapidly at low temperatures.

Figure 12 shows a comparison (on a per hydrogen atom basis) of the rate constants for abstraction from ethers at different sites. The oxygen lone pair lowers the bond dissociation energy of the α C–H bonds, which increases the rate constants at these sites. Figure 12d summarizes the rate constants for all ether abstraction sites. The α(t) carbon site (red line) has the highest abstraction rate constant, followed by the α(s) carbon site. For the tertiary carbon sites, the black and magenta lines are used to distinguish between the α and β sites. The β site shows that there is a negligible influence of the ether oxygen atom on the rate constant for abstraction; the nature of the C–H bond is similar to that in an alkane, and hence, abstraction at this site has the lowest rate constant.

Figure 13 shows comparisons of the calculated rate constants (on a per hydrogen atom basis) for abstraction from ketones at the α(p), α(s), α(t), and β(p) sites. The reaction trend for ketones is similar to that observed for ethers, in that the α(t) carbon site (red line in Figure 13d) has the highest abstraction rate constant, followed by the α(s) carbon site (blue line in Figure 13d). The rate constant for abstraction at the β(p) carbon site (magenta line) is close to that for abstraction at the α(p) carbon site (black line) at high temperatures. This reflects the fact that, as discussed earlier, the carbonyl functional group has the least effect on the energy barriers.

**Figure 14:**
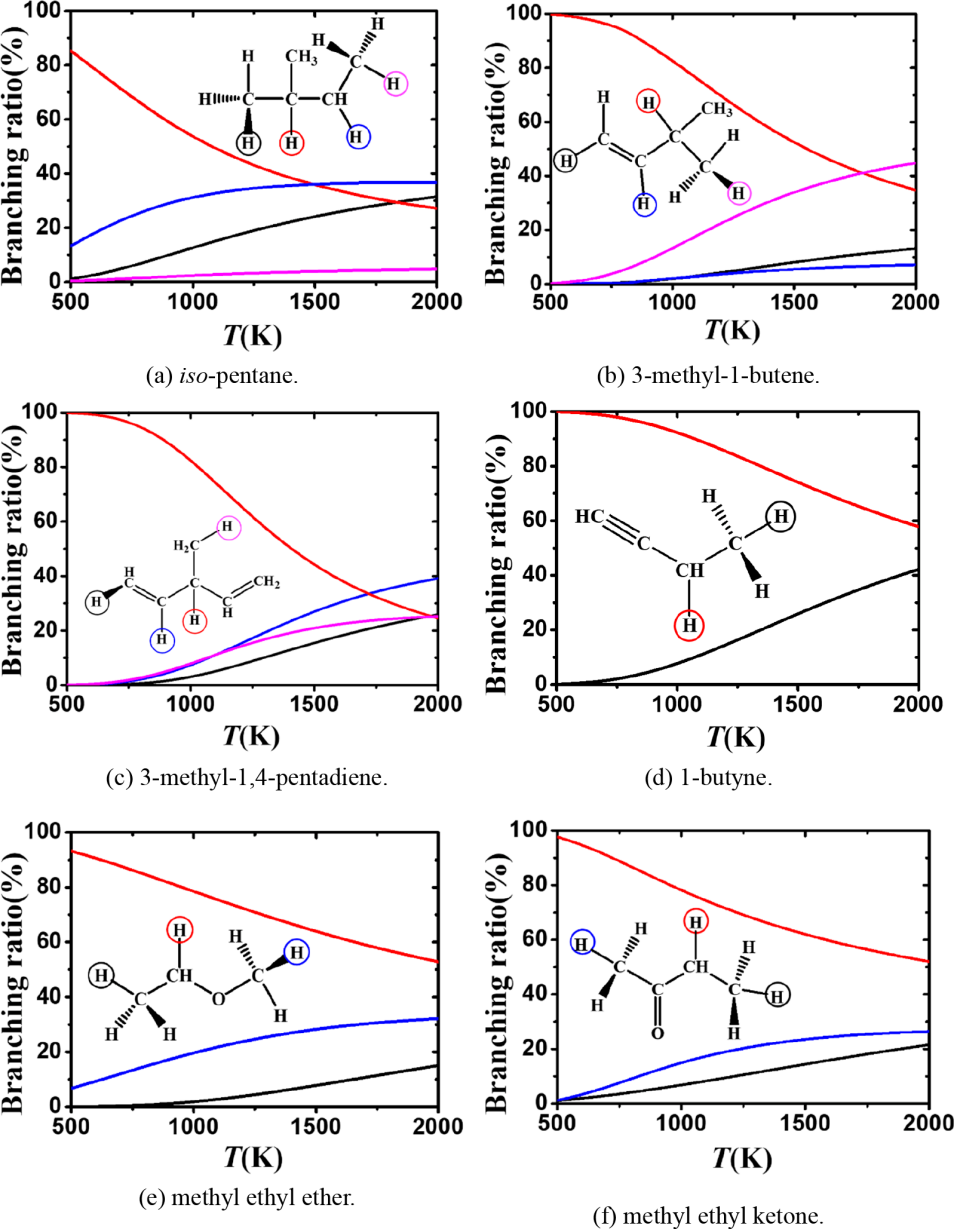
Branching ratios for abstraction by CH_3_Ȯ_2_ radicals from (a) isopentane, (b) 3-methyl-1-butene, (c) 3-methyl-1,4-pentadiene, (d) 1-butyne, (e) methyl ethyl ether, and (f) methyl ethyl ketone over the temperature range of 500–2000 K.

Figure 14 shows the branching ratios for H atom abstraction on a per site basis from iso-pentane, 3-methyl-1-butene, 3-methyl-1,4-pentadiene, 1-butyne, methyl ethyl ether, and methyl ethyl ketone. For iso-pentane (Figure 14a), the hydrogen atom on the tertiary carbon is the weakest and thus abstraction of that hydrogen atom is the fastest. However, at higher temperatures abstraction from the secondary carbon sites becomes more important. For 3-methyl-1-butene (Figure 14b), abstraction from the α position dominates in the temperature range 500–1800 K. Note that the branching ratio for abstraction at the β(p) site accounts for more than 40% of the overall rate of abstraction at temperatures above 1800 K, while abstraction from the vinylic secondary site and tertiary sites is negligible over the entire temperature range. For 3-methyl-1,4-pentadiene (Figure 14c), abstraction from the α(t) carbon site is dominant in the temperature range 500–1800 K while at higher temperatures abstraction from the tertiary vinylic carbon site becomes dominant. Figure 14d shows that for 1-butyne, the production of CH≡CĊHCH_3_ + CH_3_OOH is dominant at all temperatures investigated here. The branching ratios for abstraction from methyl ethyl ether and methyl ethyl ketone (Figure 14e,f) are consistent with the trends discussed above. Abstraction from the α(s) carbon site dominates in the temperature range 500–2000 K, while the branching ratios for abstraction from the α(p) site increases from 6.8% at 500 K to 32.1% at 2000 K for methyl ethyl ether and from 7.3% to 33.6% for methyl ethyl ketone.

#### 3.3.2 High-Pressure-Limit Rate Rules

Rate constants for all of the channels investigated in this work have been fitted by the modified Arrhenius expression *k* = *AT*^*n*^ exp(−*E*_a_/*RT*) over the temperature range of 500–2000 K, and the values of the fit parameters *A*, *n*, and *E*_a_ are provided in the Supporting Information. The average rate rules for each class are presented in Table 8.

**Table 8:** Average Rate Constant *k*(*T*) = *AT*^*n*^ exp(−*E*_a_/*RT*) for the Different Types of H-Atom Abstraction Reactions for Different Species for *T* = 500–2000 K

type	*A*	*n*	*E*_a_ (cal mol^–1^)
Alkanes + CH_3_Ȯ_2_			
primary	3.94 × 10^–6^	5.39	12344.5
secondary	2.90 × 10^–1^	4.14	15439.0
tertiary	5.00 × 10^0^	3.73	13528.7
Alkenes + CH_3_Ȯ_2_			
allylic-primary	2.41 × 10^–6^	5.43	11683.9
allylic-secondary	1.52 × 10^–4^	4.89	10232.22
allylic-tertiary	5.09 × 10^–3^	4.52	10329.7
vinylic-secondary	6.95 × 10^1^	3.41	23915.8
vinylic-tertiary	5.19 × 10^0^	3.70	20453.9
alkylic	5.60 × 10^–1^	4.03	19895.0
Dienes + CH_3_Ȯ_2_			
α-primary	2.06 × 10^–5^	5.11	9794.2
α-secondary	7.07 × 10^–3^	4.47	9675.5
α-tertiary	2.87 × 10^–4^	4.85	5642.1
β	3.82 × 10^0^	3.77	18299.7
Alkynes + CH_3_Ȯ_2_			
α-primary	5.96 × 10^–4^	4.58	11902.3
α-secondary	8.36 × 10^–3^	4.36	10601.5
α-tertiary	7.04 × 10^–2^	4.02	9414.8
β	3.02 × 10^–3^	4.66	18433.0
Ethers + CH_3_Ȯ_2_			
α-primary	6.19 × 10^–6^	5.37	12917.0
α-secondary	1.39 × 10^–2^	4.43	11608.4
α-tertiary	1.77 × 10^–4^	5.07	10583.3
β	4.66 × 10^–3^	4.49	19087.4
Ketones + CH_3_Ȯ_2_			
α-primary	2.45 × 10^–9^	6.22	13807.2
α-secondary	3.04 × 10^–5^	5.07	12959.0
α-tertiary	5.83 × 10^–6^	5.34	11158.44
β	1.72 × 10^–5^	5.04	16963.8

#### 3.3.3 Comparison of Rate Constants between Different Functional Groups at the Same Site

**Figure 15:**
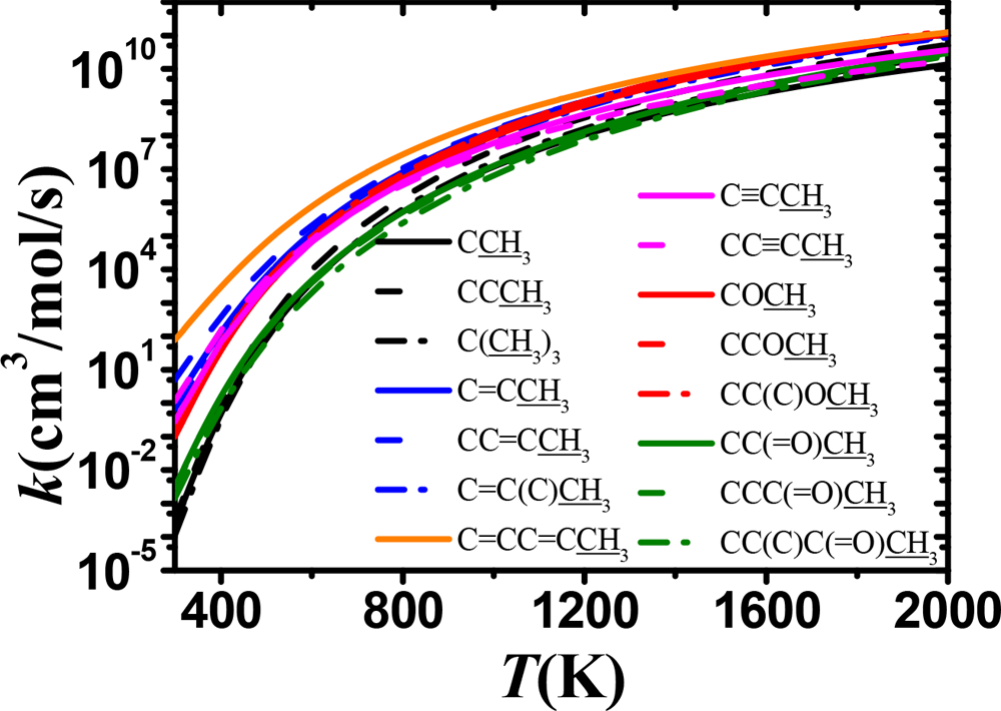
Comparison of the rate constants, on a per H atom basis, for abstraction at the α(p) site in alkanes (black), alkenes (blue), dienes (orange), alkynes (magenta), ethers (red), and ketones (green).

**Figure 16:**
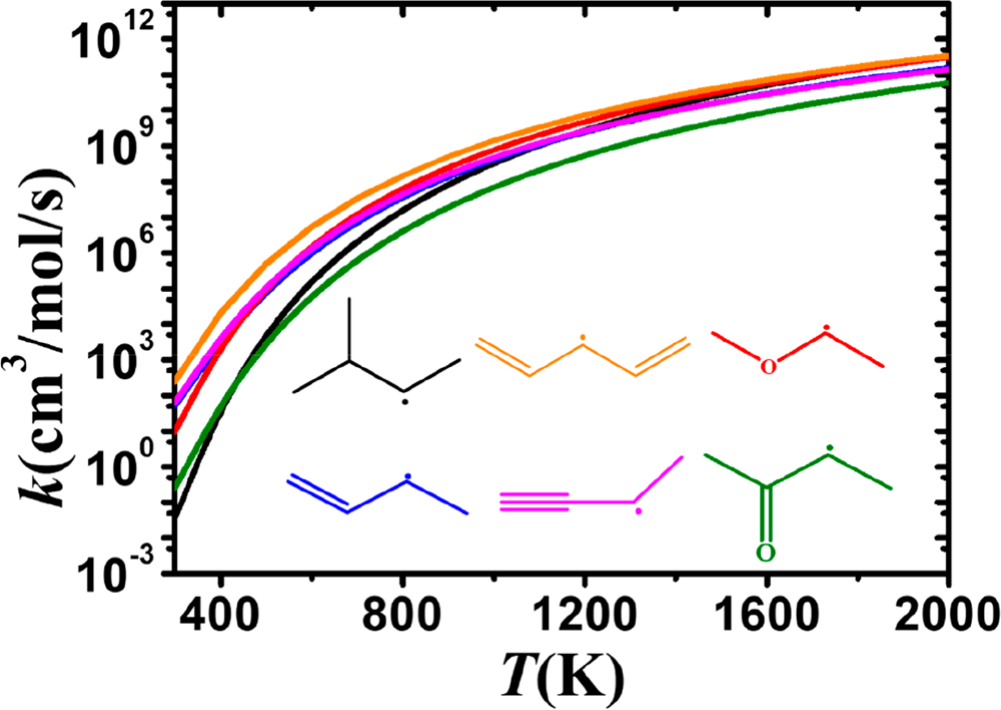
Comparison of rate constants, on a per H atom basis, for abstraction from the α(s) site in alkanes (black), alkenes (blue), dienes (orange), alkynes (magenta), ethers (red), and ketones (green).

**Figure 17:**
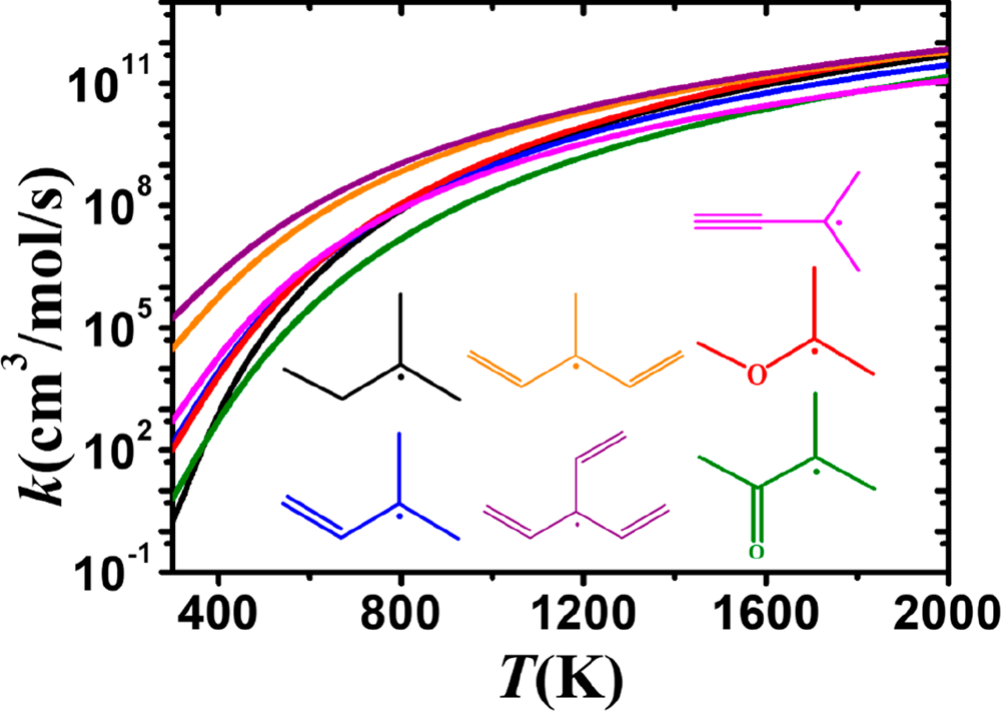
Comparison of rate constants, on a per H atom basis, for abstraction at the α(t) site in alkanes (black), alkenes (blue), dienes (orange), triene (purple), alkynes (magenta), ethers (red), and ketones (green).

**Figure 18:**
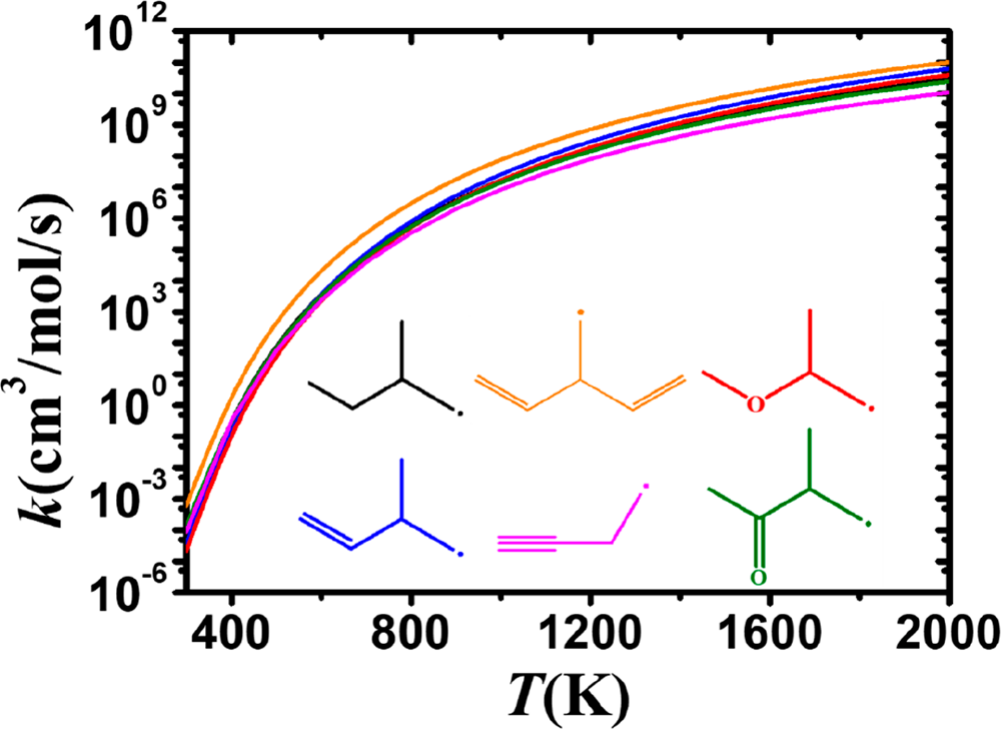
Comparison of the rate constants, on a per H atom basis, for abstraction from the β site in alkanes (black), alkenes (blue), dienes (orange), alkynes (magenta), ethers (red), and ketones (green).

In this work, we use alkane fuels as a benchmark and compare the rate constants for abstraction at similar sites from alkenes, dienes, trienes, alkynes, ethers, and ketones, all of which contain different functional groups. Comparisons of the rate constants, including abstraction from the α(p), α(s), α(t), and β(p) carbon sites are presented in Figures 15–18.

Figure 15 shows a comparison of the rate constants, on a per hydrogen atom basis, for abstraction from alkanes, alkenes, dienes, alkynes, ethers, and ketones at the α(p) position. Abstraction from 1,3-pentadiene is the fastest, and at 500 K the calculated rate constant is 522 and 391 times higher than the lowest ones for isopropyl methyl ketone and iso-pentane, respectively. At 2000 K, this difference decreases to factors of approximately 4.2 and 3.0, respectively. For alkenes, the rate constants are very close to alkanes at high temperatures (2000 K) but are about a factor of 9.5 higher at 500 K. There is little difference observed in the rate constants for abstraction from monoalkenes, ethers, and alkynes, which have lower energy barriers compare to alkanes, with the rate constants being 1–2 orders of magnitude higher compared to alkanes in the temperature range 300–1000 K. The carbonyl group in ketones has the smallest effect on the rate constants. At higher temperatures, the influence of the functional group diminishes, and the rate gap decreases.

The same trend is observed for all hydrogen atoms located at α(s) and α(t) sites, for which the calculated rate constants are presented in Figures 16 and 17, respectively. In order to present the rate constants more clearly, we have selected one species to represent each functional group for comparison. Figure 16 shows that abstraction at the α(s) site from 1,4-pentadiene has the highest rate constant at all temperatures. The effect of the functional group on the calculated rate constant for 1-butene, 1-butyne, and methyl ether is also relatively large, and these rate constants are significantly higher compared to those for abstraction from iso-pentane. We also find that the presence of the carbonyl group lowers the rate constant compared to the corresponding alkane. Figure 17 illustrates the rate constants on a per hydrogen atom basis for abstraction from the α(t) carbon site. It is evident that abstraction from the tertiary carbon sites in 3-vinyl-1,4-pentadiene and 3-methyl-1,4-pentadiene have the highest rate constants, indicating that the triene and diene functional groups have a strong influence on the rate constant, which is related to their lower energy barriers. In particular, the rate constant for 3-vinyl-1,4-pentadiene is similar to those for 3-methyl-1-butene and iso-pentane at 2000 K and is higher by factors of 71 and 232, respectively, at 500 K.

The results presented in Figures 16 and 17 demonstrate that the functional group in alkenes, alkynes, and ethers will promote the rate constants at the α(s) and α(t) sites compared to alkanes at temperatures below 1200 and 800 K, respectively. There is little difference observed in rate constant for abstraction from the β site as shown inFigure 18, except for 3-methyl-1,4-pentadiene, at which point the effect of the functional group becomes very weak. For 3-methyl-1,4-pentadiene, the influence of the functional group decreases significantly at high temperatures.

### 3.4 Inter-rotational Influence from the Functional Hindrance Potential

From Figures 16 and 17, it can be concluded that the influence on the rate of abstraction of the −C=O functional group is much weaker than the other functional groups. An internal hindered rotor treatment has been performed to explore the difference in functional group effect for ketones and ethers. We have chosen two oxygenated functional group systems, ketone and ether, to compare the performance of internal hindered rotor potentials to further explore the effect of functional groups on the rate constants.

**Figure 19:**
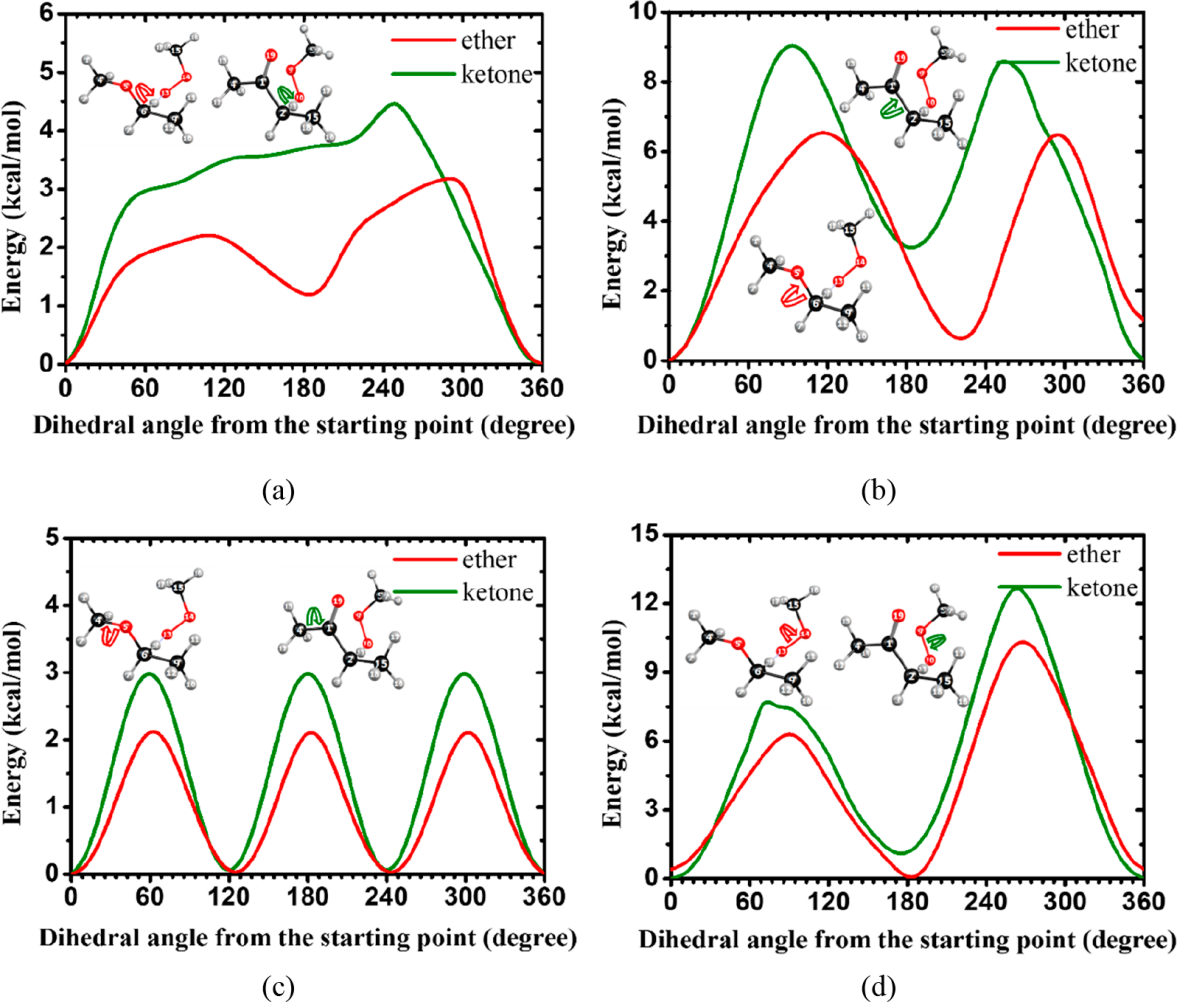
Rotational potentials for the same C–O and C–C torsional modes in the TSs obtained at the M06-2X/6-31G level of theory for the ethyl methyl ether + CH_3_Ȯ_2_ reaction (red lines) and the ethyl methyl ketone + CH_3_Ȯ_2_ reaction (green lines).

Apart from different geometries and electronic energy barriers, the partition functions for the transition states, calculated with the RRHO treatment for vibrational frequencies and the 1D hindrance potential, are also quite different. Moreover, different rotational–vibrational properties corresponding to the different TS geometries also contributes to the difference in rate constants. Figure 19 shows the hindrance potentials for C–O and C–C torsional modes at similar position of the TSs for the ethyl methyl ether + CH_3_Ȯ_2_ and ethyl methyl ketone + CH_3_Ȯ_2_ reactions. Figure 19a shows that the hindrance potential of the C–O bond in methyl ethyl ketone is 1–2 kcal mol^–1^ higher than that in methyl ethyl ether, which reduces their contribution to the hindrance partition function and further decreases the rate constant. Figure 19b,c shows the difference of rotational potentials of C–O bond in ethers and C–C bond in ketones. The same trend can be observed, with the hindrance potential of ketones being higher than that of ethers, which would further decrease the rate constants. In Figure 19d, the influence of the functional group on the torsional potential of the O–O bond decreases, and the torsional potentials of the two transition states are similar; however, the potential of the ketone still has a higher energy barrier than that of the ether. These all cause the final rate constants for ketones to be lower than those in ethers at the α(s) site.

**Figure 20:**
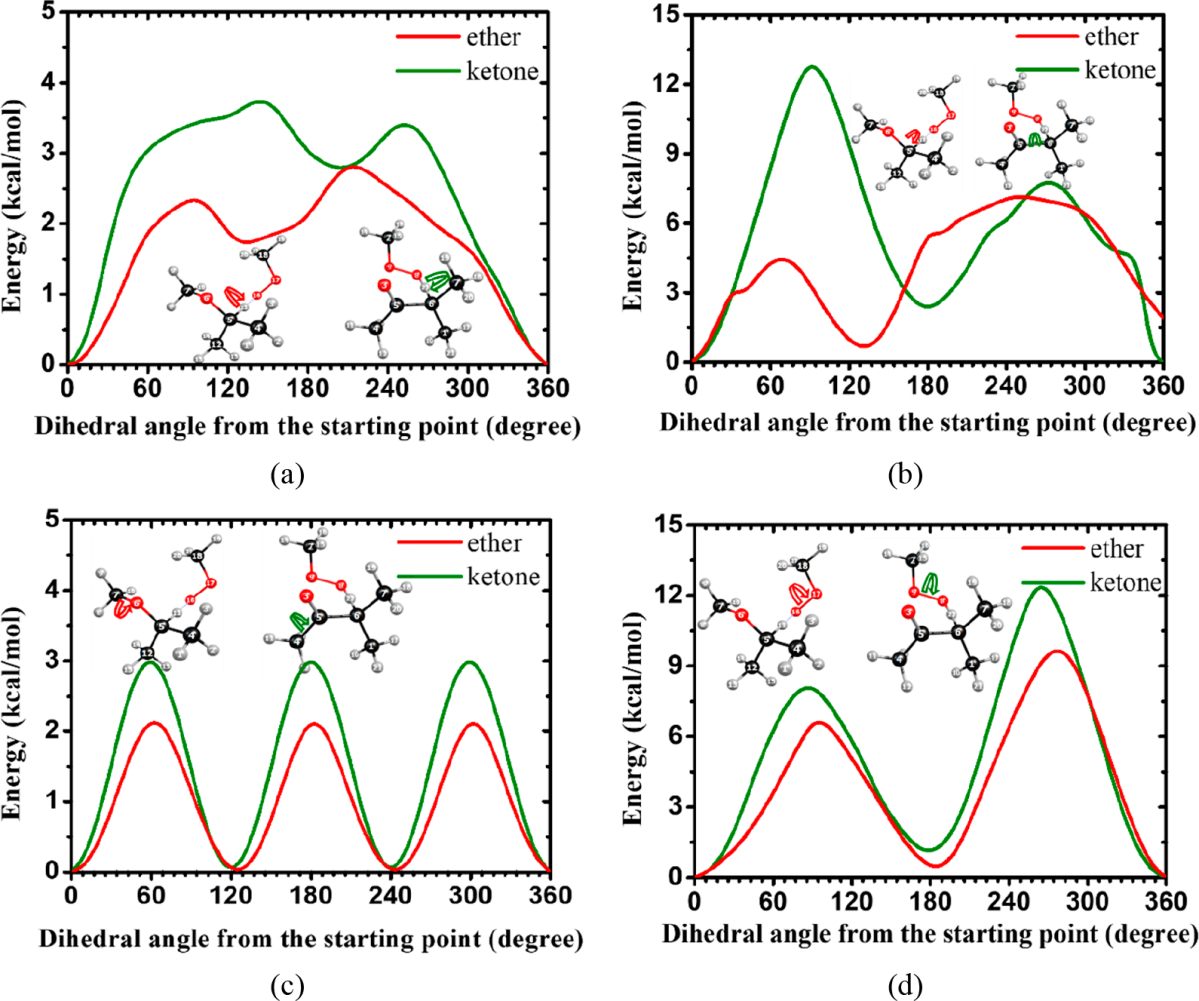
Rotational potentials for the same C–O and C–C torsional modes in the TSs obtained at the M06-2X/6-31G level of theory for the H atom abstraction by CH_3_Ȯ_2_ at the α(t) site of isopropyl methyl ether (red lines) and isopropyl methyl ketone (green lines).

Figure 20 illustrates the rotational potentials of C–O and C–C torsional modes at the similar positions of the TSs for the isopropyl methyl ether + CH_3_Ȯ_2_ and isopropyl methyl ketone + CH_3_Ȯ_2_ reactions. As shown in Figure 20a, the torsional potential of the C–O bond in the transition state is ∼1 kcal mol^–1^ higher for the ketone than for the ether. Similar trends were found in other positions, as shown in Figure 20b–d, with a higher rotational potential for the isopropyl methyl ketone + CH_3_Ȯ_2_ TS, which reduces the hindrance partition function and gives a lower rate constant than that for the isopropyl methyl ether + CH_3_Ȯ_2_ reaction.

## 4 Conclusions

We have presented a systematic study of the kinetics of hydrogen atom abstraction reactions by CH_3_Ȯ_2_ radicals from species of alkanes, alkenes, dienes, alkynes, ethers, and ketones with different functional groups. Electronic structure optimization, frequency analysis, and zero-point energy corrections were performed for 26 reactants, 61 transition states, and 62 products under the approach of quantum chemistry at the M06-2X/6-311++G(d,p) level. Single-point energy calculations were performed at the QCISD(T)/CBS level of theory. Temperature-dependent rate constants were calculated for all reaction systems in the temperature range of 298.15–2000 K using transition state theory, including corrections for asymmetric tunneling effects.

The calculations show that the energy barrier is lower for H atom abstraction from the site α to the functional group, while that effect decreases pronouncedly for the β site. The rate constants trends are consistent with the calculated electronic energy barriers. The presence of functional groups has the largest effect on the α-primary position followed by the α-secondary and α-tertiary positions.

For species containing different functional groups, the dienes and trienes have the largest effect on the rate constant investigated. Carbon–carbon triple bonds (C≡C) and ether groups (−O−) also can increase the reaction rate constants at the α sites, which are significantly higher compared to those for alkanes. The carbonyl functional group does not have a strong influence on the rate constant at the α position. This work provides detailed rate constants for the reactions of CH_3_Ȯ_2_ radicals with fuels containing different functional groups, which can be used in the development of combustion models for these fuels.

## Supporting Information Available

T1 diagnostic values and fitted values of the parameters in the modified Arrhenius expression for rate constant calculations on a per site basis (PDF)
Fitted values of the parameters in the modified Arrhenius expression and mean and maximum errors of the fits (PDF)
