# Supplementary material for: Kinetic Properties Study of H Atom Abstraction by CH3Ȯ2 Radicals from Fuel Molecules with Different Functional Groups
Source: J Phys Chem A. 2023 Feb 20;127(8):1960–74. doi: 10.1021/acs.jpca.2c08100 (PMC9986872; doi:10.1021/acs.jpca.2c08100)
Supplement: Supplementary file 1 — jp2c08100_si_001.pdf [file jp2c08100_si_001.pdf]

Supporting Information

Kinetic Properties Study of H-atom Abstraction by $CH_3\dot{O}_2$ Radicals from Fuel Molecules with Different Functional Groups

Hao-Ting Guo[1], Yan Tang[1], Sheng-Han Liu[1], Yang Ma[1], Shen Fang[1], Henry J. Curran[2], Chong-Wen Zhou[1,2*]

[1]*School of Energy and Power Engineering, Beihang University, Beijing 100191*

[2]*Combustion Chemistry Centre, School of Biological and Chemical Sciences, Ryan Institute, University of Galway, Galway H91TK33, Ireland*

E-mail address: chongwen.zhou@universityofgalway.ie (C.-W. Zhou)

When calculating the energy using the QCISD(T) method care should be taken to observe the T1 diagnostic values to measure the multi-reference state effect. Table S1 lists the T1 diagnostic values for all the reaction transition states studied in this paper, and all T1 diagnostic values are less than 0.035.

Table S1. T1 diagnostic values for the transition state of the H-atom abstraction reaction by CH_3O_2 obtained under the QCISD(T) method

No.	Reaction Channel	T1 diagnostic
R1	CC→CĊ	0.02
R2	CCC→CĊC	0.02
R3	CCC→ĊCC	0.02
R4	CC(C)C→ĊC(C)C	0.02
R5	CC(C)C→CĊ(C)C	0.02
R6	CC(C)CC→ĊC(C)CC	0.02
R7	CC(C)CC→CĊ(C)CC	0.02
R8	CC(C)CC→CC(C)ĊC	0.02
R9	CC(C)CC→CC(C)CĊ	0.02
R10	C=C→Ċ=C	0.02
R11	C=CC→Ċ=CC	0.03
R12	C=CC→C=ĊC	0.03
R13	C=CC→C=CĊ	0.03
R14	C=CCC→Ċ=CCC	0.03
R15	C=CCC→C=ĊCC	0.03
R16	C=CCC→C=CĊC	0.03
R17	C=CCC→C=CCĊ	0.03
R18	CC=CC→ĊC=CC	0.03
R19	CC=CC→CĊ=CC	0.03
R20	C=C(C)C→Ċ=C(C)C	0.03
R21	C=C(C)C→C=C(C)Ċ	0.03
R22	C=CC(C)C→Ċ=CC(C)C	0.03
R23	C=CC(C)C→C=ĊC(C)C	0.03
R24	C=CC(C)C→C=CĊ(C)C	0.03
R25	C=CC(C)C→C=CC(C)Ċ	0.03
R26	C=CC=C→Ċ=CC=C	0.03
R27	C=CC=C→C=ĊC=C	0.03
R28	C=CC=CC→Ċ=CC=CC	0.03
R29	C=CC=CC→C=ĊC=CC	0.03
R30	C=CC=CC→C=CĊ=CC	0.03
R31	C=CC=CC→C=CC=ĊC	0.03
R32	C=CC=CC→C=CC=CĊ	0.03
R33	C=CCC=C→Ċ=CCC=C	0.03
R34	C=CCC=C→C=ĊCC=C	0.03
R35	C=CCC=C→C=CĊC=C	0.03
R36	C=CC(C)C=C→Ċ=CC(C)C=C	0.03

R37	C=CC(C)C=C→C=ĊC(C)C=C	0.03
R38	C=CC(C)C=C→C=CĊ(C)C=C	0.03
R39	C=CC(C)C=C→C=CC(Ċ)C=C	0.03
R40	C=CC(C=C)C=C→Ċ=CC(C=C)C=C	0.02
R41	C=CC(C=C)C=C→C=ĊC(C=C)C=C	0.02
R42	C=CC(C=C)C=C→C=CĊ(C=C)C=C	0.02
R43	C≡CC→C≡CĊ	0.02
R44	CC≡CC→CC≡CĊ	0.03
R45	C≡CCC→C≡CĊC	0.03
R46	C≡CCC→C≡CCĊ	0.03
R47	C≡CC(C)C→C≡CĊ(C)C	0.03
R48	COC→ĊOC	0.02
R49	COCC→ĊOCC	0.02
R50	COCC→COĊC	0.02
R51	COCC→COCĊ	0.03
R52	COC(C)C→ĊOC(C)C	0.02
R53	COC(C)C→COĊ(C)C	0.03
R54	COC(C)C→COC(C)Ċ	0.02
R55	CC(=O)C→ĊC(=O)C	0.02
R56	CC(=O)CC→ĊC(=O)CC	0.02
R57	CC(=O)CC→CC(=O)ĊC	0.02
R58	CC(=O)CC→CC(=O)CĊ	0.02
R59	CC(=O)C(C)C→ĊC(=O)C(C)C	0.03
R60	CC(=O)C(C)C→CC(=O)Ċ(C)C	0.02
R61	CC(=O)C(C)C→CC(=O)C(C)Ċ	0.02

The H-atom abstraction reaction rate constants of CH_3O_2 radicals for different types of reactants at each reaction site were fitted by the three-parameter A, n, Ea modified Arrhenius equation $k = A \times T^n \times exp(-E_a/RT)$, and the values of the fitted A, n, E_a parameters are listed in Table S2.

Table S2. The modified Arrhenius expressions for rate constants of abstracting H-atom by CH_3O_2 radical at each reaction site on per site basis. ($k(T) = AT^n exp(-E_a/RT)$. T = 500–2000 K).

No.	Reaction Channel	A	n	E_a (cal mol^{-1})
R1	CC→CĊ	8.15E+00	3.76E+00	1.76E+04
R2	CCC→CĊC	8.43E+00	3.74E+00	1.54E+04
R3	CCC→ĊCC	4.99E+00	3.88E+00	1.83E+04
R4	CC(C)C→ĊC(C)C	4.85E+00	3.85E+00	1.85E+04
R5	CC(C)C→CĊ(C)C	1.59E+01	3.47E+00	1.31E+04
R6	CC(C)CC→ĊC(C)CC	2.71E+00	4.08E+00	1.94E+04
R7	CC(C)CC→CĊ(C)CC	1.13E+01	3.69E+00	1.41E+04
R8	CC(C)CC→CC(C)ĊC	4.28E-01	4.23E+00	1.60E+04
R9	CC(C)CC→CC(C)CĊ	2.63E-01	4.10E+00	1.83E+04
R10	C=C→Ċ=C	9.88E+00	3.73E+00	2.35E+04
R11	C=CC→Ċ=CC	2.86E+01	3.58E+00	2.44E+04

R12	C=CC→C=ĊC	8.94E+01	3.25E+00	2.06E+04
R13	C=CC→C=CĊ	4.35E-05	5.24E+00	1.25E+04
R14	C=CCC→Ċ=CCC	1.02E+02	3.45E+00	2.40E+04
R15	C=CCC→C=ĊCC	4.95E+00	3.77E+00	2.05E+04
R16	C=CCC→C=CĊC	3.03E-04	4.89E+00	1.02E+04
R17	C=CCC→C=CCĊ	3.75E+00	3.95E+00	2.01E+04
R18	CC=CC→ĊC=CC	5.53E-05	5.23E+00	1.13E+04
R19	CC=CC→CĊ=CC	9.25E+00	3.66E+00	2.05E+04
R20	C=C(C)C→Ċ=C(C)C	1.01E+05	2.54E+00	2.46E+04
R21	C=C(C)C→C=C(C)Ċ	3.04E-06	5.63E+00	1.21E+04
R22	C=CC(C)C→Ċ=CC(C)C	6.86E+01	3.58E+00	2.38E+04
R23	C=CC(C)C→C=ĊC(C)C	3.05E+00	3.80E+00	2.04E+04
R24	C=CC(C)C→C=CĊ(C)C	5.09E-03	4.52E+00	1.03E+04
R25	C=CC(C)C→C=CC(C)Ċ	1.23E+00	4.13E+00	1.96E+04
R26	C=CC=C→Ċ=CC=C	2.51E+02	3.56E+00	2.56E+04
R27	C=CC=C→C=ĊC=C	7.31E+02	3.43E+00	2.07E+04
R28	C=CC=CC→Ċ=CC=CC	4.00E+04	2.86E+00	2.65E+04
R29	C=CC=CC→C=ĊC=CC	2.07E+03	3.13E+00	2.06E+04
R30	C=CC=CC→C=CĊ=CC	1.37E+04	2.92E+00	2.30E+04
R31	C=CC=CC→C=CC=ĊC	1.16E+02	3.53E+00	2.23E+04
R32	C=CC=CC→C=CC=CĊ	6.17E-05	5.11E+00	9.79E+03
R33	C=CCC=C→Ċ=CCC=C	1.30E+02	3.71E+00	2.49E+04
R34	C=CCC=C→C=ĊCC=C	7.87E+00	3.91E+00	2.13E+04
R35	C=CCC=C→C=CĊC=C	1.42E-02	4.47E+00	9.68E+03
R36	C=CC(C)C=C→Ċ=CC(C)C=C	2.19E+03	3.34E+00	2.35E+04
R37	C=CC(C)C=C→C=ĊC(C)C=C	4.76E+02	3.41E+00	1.98E+04
R38	C=CC(C)C=C→C=CĊ(C)C=C	4.57E-03	4.51E+00	6.89E+03
R39	C=CC(C)C=C→C=CC(Ċ)C=C	2.29E+01	3.77E+00	1.83E+04
R40	C=CC(C=C)C=C→Ċ=CC(C=C)C=C	7.80E+03	3.24E+00	2.33E+04
R41	C=CC(C=C)C=C→C=ĊC(C=C)C=C	8.41E+02	3.38E+00	2.01E+04
R42	C=CC(C=C)C=C→C=CĊ(C=C)C=C	1.18E-04	4.95E+00	5.05E+03
R43	C≡CC→C≡CĊ	2.71E-03	4.48E+00	1.32E+04
R44	CC≡CC→CC≡CĊ	8.45E-03	4.52E+00	1.18E+04
R45	C≡CCC→C≡CĊC	1.67E-02	4.36E+00	1.06E+04
R46	C≡CCC→C≡CCĊ	9.05E-03	4.66E+00	1.84E+04
R47	C≡CC(C)C→C≡CĊ(C)C	7.04E-02	4.02E+00	9.41E+03
R48	COC→ĊOC	1.56E-05	5.47E+00	1.30E+04
R49	COCC→ĊOCC	6.10E-05	5.22E+00	1.30E+04
R50	COCC→COĊC	2.78E-02	4.43E+00	1.16E+04
R51	COCC→COCĊ	7.42E-04	5.02E+00	2.00E+04
R52	COC(C)C→ĊOC(C)C	1.07E-05	5.45E+00	1.27E+04

R53	COC(C)C→COĊ(C)C	1.77E-04	5.07E+00	1.06E+04
R54	COC(C)C→COC(C)Ċ	8.39E-01	4.13E+00	1.97E+04
R55	CC(=O)C→ĊC(=O)C	8.34E-08	6.02E+00	1.40E+04
R56	CC(=O)CC→ĊC(=O)CC	7.19E-09	6.21E+00	1.36E+04
R57	CC(=O)CC→CC(=O)ĊC	6.09E-05	5.07E+00	1.30E+04
R58	CC(=O)CC→CC(=O)CĊ	6.34E-12	7.03E+00	1.49E+04
R59	CC(=O)C(C)C→ĊC(=O)C(C)C	4.72E-10	6.55E+00	1.38E+04
R60	CC(=O)C(C)C→CC(=O)Ċ(C)C	5.83E-06	5.34E+00	1.12E+04
R61	CC(=O)C(C)C→CC(=O)C(C)Ċ	1.11E-01	4.19E+00	1.81E+04
