# Supplementary material for: Kinetic Properties Study of H Atom Abstraction by CH3Ȯ2 Radicals from Fuel Molecules with Different Functional Groups
Source: J Phys Chem A. 2023 Feb 20;127(8):1960–74. doi: 10.1021/acs.jpca.2c08100 (PMC9986872; doi:10.1021/acs.jpca.2c08100)
Supplement: Supplementary file 2 — jp2c08100_si_002.pdf [file jp2c08100_si_002.pdf]

Kinetic Properties Study of H-atom Abstraction by $CH_3\dot{O}_2$ Radicals from Fuel Molecules with Different Functional Groups

Hao-Ting Guo[1], Yan Tang[1], Sheng-Han Liu[1], Yang Ma[1], Shen Fang[1],
Henry J. Curran[2], Chong-Wen Zhou[1,2*]

[1]School of Energy and Power Engineering, Beihang University, Beijing 100191

[2]Combustion Chemistry Centre, School of Biological and Chemical Sciences, Ryan Institute, University of Galway, Galway H91TK33, Ireland

E-mail address: cwzhou@buaa.edu.cn (C.-W. Zhou)

The H-atom abstraction reaction rate constants of CH_3O_2 radicals for different types of reactants at each reaction site were fitted by the three-parameter A, n, Ea modified Arrhenius equation $k = A \times T^n \times exp(-E_a/RT)$, and the values of the fitted A, n, E_a parameters, mean error and max error are provided.

! H-atom abstraction reactions of alkanes + CH_3O_2

$CH_3CH_3 + CH_3O_2 = CH_3CH_2 + CH_3OOH$ +8.14742000E+00 +3.76151000E+00 +1.76173712E+04 ! Fitting from 500.00 to 2000.00 K, Mean Error = 1.65 % Max Error = 3.29 %

$CH_3CH_2CH_3 + CH_3O_2 = CH_3CH_2CH_2 + CH_3OOH$ +4.99230000E+00 +3.87921000E+00 +1.82520380E+04 !Fitting from 500.00 to 2000.00 K, Mean Error = 1.82 % Max Error = 3.53 %

$CH_3CH_2CH_3 + CH_3O_2 = CH_3CHCH_3 + CH_3OOH$ +8.43480000E+00 +3.74158000E+00 +1.54319486E+04 ! Fitting from 500.00 to 2000.00 K, Mean Error = 1.63 % Max Error = 3.28 %

$CH_3CH(CH_3)_2 + CH_3O_2 = CH_3C(CH_3)_2 + CH_3OOH$ +1.58607500E+01 +3.47309000E+00 +1.31385274E+04 ! Fitting from 500.00 to 2000.00 K, Mean Error = 1.27 % Max Error = 2.53 %

$CH_3CH(CH_3)_2 + CH_3O_2 = CH_2CH(CH_3)_2 + CH_3OOH$ +4.84982000E+00 +3.84616000E+00 +1.84803498E+04 ! Fitting from 500.00 to 2000.00 K, Mean Error = 1.79 % Max Error = 3.59 %

$CH_3CH(CH_3)CH_2CH_3 + CH_3O_2 = CH_2CH(CH_3)CH_2CH_3 + CH_3OOH$ +2.71111000E+00 +4.07692000E+00 +1.93603750E+04 ! Fitting from 500.00 to 2000.00 K, Mean Error = 2.10 % Max Error = 4.16 %

$CH_3CH(CH_3)CH_2CH_3 + CH_3O_2 = CH_3C(CH_3)CH_2CH_3 + CH_3OOH$ +1.13289300E+01 +3.69281000E+00 +1.40642330E+04 ! Fitting from 500.00 to 2000.00 K, Mean Error = 1.70 % Max Error = 3.49 %

$CH_3CH(CH_3)CH_2CH_3 + CH_3O_2 = CH_3CH(CH_3)CHCH_3 + CH_3OOH$ +4.27580000E-01 +4.22861000E+00 +1.59758417E+04 ! Fitting from 500.00 to 2000.00 K, Mean Error = 2.38 % Max Error = 4.68 %

$CH_3CH(CH_3)CH_2CH_3 + CH_3O_2 = CH_3CH(CH_3)CH_2CH_2 + CH_3OOH$ +2.63400000E-01 +4.10078000E+00 +1.83480128E+04 ! Fitting from 500.00 to 2000.00 K, Mean Error = 2.18 % Max Error = 4.34 %

! H-atom abstraction reactions of alkenes + CH_3O_2

$CH_2=CH_2 + CH_3O_2 = CH_2=CH + CH_3OOH$ +9.87879000E+00 +3.73335000E+00 +2.35139021E+04 ! Fitting from 500.00 to 2000.00 K, Mean Error = 1.44 % Max Error = 2.93 %

$CH_2=CHCH_3 + CH_3O_2 = CH=CHCH_3 + CH_3OOH$ +2.85645100E+01 +3.58299000E+00 +2.43911822E+04 ! Fitting from 500.00 to 2000.00 K,

Mean Error = 1.33 % Max Error = 2.47 %

CH_2=$CHCH_3$ + CH_3O_2 = CH_2=CCH_3 + CH_3OOH +8.94296200E+01 +3.25105000E+00 +2.05929894E+04 ! Fitting from 500.00 to 2000.00 K, Mean Error = 1.48 % Max Error = 2.89 %

CH_2=$CHCH_3$ + CH_3O_2 = CH_2=$CHCH_2$ + CH_3OOH +4.34528000E-05 +5.24012000E+00 +1.24984109E+04 ! Fitting from 500.00 to 2000.00 K, Mean Error = 3.50 % Max Error = 7.11 %

CH_2=$C(CH_3)_2$ + CH_3O_2 = CH=$C(CH_3)_2$ + CH_3OOH +1.00860036E+05 +2.54230000E+00 +2.45704996E+04 ! Fitting from 500.00 to 2000.00 K, Mean Error = 1.25 % Max Error = 2.73 %

CH_2=$C(CH_3)_2$ + CH_3O_2 = CH_2=$C(CH_3)CH_2$ + CH_3OOH +3.04324000E-06 +5.63077000E+00 +1.20787704E+04 ! Fitting from 500.00 to 2000.00 K, Mean Error = 3.94 % Max Error = 8.55 %

CH_2=$CHCH_2CH_3$ + CH_3O_2 = CH=$CHCH_2CH_3$ + CH_3OOH +1.01813360E+02 +3.45182000E+00 +2.39693196E+04 ! Fitting from 500.00 to 2000.00 K, Mean Error = 1.10 % Max Error = 2.28 %

CH_2=$CHCH_2CH_3$ + CH_3O_2 = CH_2=CCH_2CH_3 + CH_3OOH +4.94893000E+00 +3.77403000E+00 +2.05274258E+04 ! Fitting from 500.00 to 2000.00 K, Mean Error = 1.61 % Max Error = 3.14 %

CH_2=$CHCH_2CH_3$ + CH_3O_2 = CH_2=$CHCHCH_3$ + CH_3OOH +3.03327000E-04 +4.89398000E+00 +1.02322183E+04 ! Fitting from 500.00 to 2000.00 K, Mean Error = 3.01 % Max Error = 6.10 %

CH_2=$CHCH_2CH_3$ + CH_3O_2 = CH_2=$CHCH_2CH_2$ + CH_3OOH +3.74687000E+00 +3.95088000E+00 +2.01047070E+04 ! Fitting from 500.00 to 2000.00 K, Mean Error = 1.68 % Max Error = 3.55 %

$CH_3CH{=}CHCH_3 + CH_3O_2 = CH_2CH{=}CHCH_3 + CH_3OOH$ +5.52721000E-05 +5.23382000E+00 +1.12745966E+04 ! Fitting from 500.00 to 2000.00 K, Mean Error = 3.37 % Max Error = 6.85 %

$CH_3CH{=}CHCH_3 + CH_3O_2 = CH_3C{=}CHCH_3 + CH_3OOH$ +9.24851000E+00 +3.65506000E+00 +2.05333485E+04 ! Fitting from 500.00 to 2000.00 K, Mean Error = 1.42 % Max Error = 2.67 %

$CH_2{=}CHCH(CH_3)_2 + CH_3O_2 = CH{=}CHCH(CH_3)_2 + CH_3OOH$ +6.85522000E+01 +3.58150000E+00 +2.38409674E+04 ! Fitting from 500.00 to 2000.00 K, Mean Error = 1.37 % Max Error = 2.95 %

$CH_2{=}CHCH(CH_3)_2 + CH_3O_2 = CH_2{=}CCH(CH_3)_2 + CH_3OOH$ +3.04725000E+00 +3.79906000E+00 +2.04386130E+04 ! Fitting from 500.00 to 2000.00 K, Mean Error = 1.57 % Max Error = 3.07 %

$CH_2{=}CHCH(CH_3)_2 + CH_3O_2 = CH_2{=}CHC(CH_3)_2 + CH_3OOH$ +5.09000000E-03 +4.51537000E+00 +1.03296636E+04 ! Fitting from 500.00 to 2000.00 K, Mean Error = 2.85 % Max Error = 5.84%

$CH_2{=}CHCH(CH_3)_2 + CH_3O_2 = CH_2{=}CHCH(CH_3)CH_2 + CH_3OOH$ +1.22708000E+00 +4.13367000E+00 +1.96266363E+04 ! Fitting from 500.00 to 2000.00 K, Mean Error = 2.12 % Max Error = 4.18 %

! H-atom abstraction reactions of dienes + CH_3O_2

$CH_2{=}CHCH{=}CH_2 + CH_3O_2 = CH{=}CHCH{=}CH_2 + CH_3OOH$ +2.51179440E+02 +3.56388000E+00 +2.55931563E+04 ! Fitting from 500.00 to 2000.00 K, Mean Error = 1.36 % Max Error = 2.53 %

$CH_2{=}CHCH{=}CH_2 + CH_3O_2 = CH_2{=}CCH{=}CH_2 + CH_3OOH$ +7.30666640E+02 +3.43152000E+00 +2.06788262E+04 ! Fitting from 500.00 to 2000.00 K, Mean Error = 1.58 % Max Error = 3.35 %

CH_2=CHCH=CHCH$_3$ + CH_3O_2 = CH=CHCH=CHCH$_3$ + CH_3OOH +4.00357116E+04 +2.86286000E+00 +2.65167281E+04 ! Fitting from 500.00 to 2000.00 K, Mean Error = 1.32 % Max Error = 2.92 %

CH_2=CHCH=CHCH$_3$ + CH_3O_2 = CH_2=CCH=CHCH$_3$ + CH_3OOH +2.06514856E+03 +3.13494000E+00 +2.06421787E+04 ! Fitting from 500.00 to 2000.00 K, Mean Error = 1.86 % Max Error = 4.09 %

CH_2=CHCH=CHCH$_3$ + CH_3O_2 = CH_2=CHC=CHCH$_3$ + CH_3OOH +1.36973318E+04 +2.91836000E+00 +2.29921336E+04 ! Fitting from 500.00 to 2000.00 K, Mean Error = 1.90 % Max Error = 5.51 %

CH_2=CHCH=CHCH$_3$ + CH_3O_2 = CH_2=CHCH=CCH$_3$ + CH_3OOH +1.16276610E+02 +3.53056000E+00 +2.23433072E+04 ! Fitting from 500.00 to 2000.00 K, Mean Error = 1.80 % Max Error = 3.71 %

CH_2=CHCH=CHCH$_3$ + CH_3O_2 = CH_2=CHCH=CHCH$_2$ + CH_3OOH +6.17263000E-05 +5.11273000E+00 +9.79415508E+03 ! Fitting from 500.00 to 2000.00 K, Mean Error = 3.52 % Max Error = 7.35 %

CH_2=CHCH$_2$CH=CH$_2$ + CH_3O_2 = CH=CHCH$_2$CH=CH$_2$ + CH_3OOH +1.29974680E+02 +3.70547000E+00 +2.48656067E+04 ! Fitting from 500.00 to 2000.00 K, Mean Error = 1.90 % Max Error = 5.61 %

CH_2=CHCH$_2$CH=CH$_2$ + CH_3O_2 = CH_2=CCH$_2$CH=CH$_2$ + CH_3OOH +7.87013000E+00 +3.90722000E+00 +2.12718881E+04 ! Fitting from 500.00 to 2000.00 K, Mean Error = 1.55 % Max Error = 3.00 %

CH_2=CHCH$_2$CH=CH$_2$ + CH_3O_2 = CH_2=CHCHCH=CH$_2$ + CH_3OOH +1.41500000E-02 +4.47082000E+00 +9.67547909E+03 ! Fitting from 500.00 to 2000.00 K, Mean Error = 2.22 % Max Error = 4.40 %

CH_2=CHCH(CH_3)CH=CH$_2$ + CH_3O_2 = CH=CHCH(CH_3)CH=CH$_2$ + CH_3OOH +2.19488303E+03 +3.34123000E+00 +2.34547004E+04 ! Fitting from

500.00 to 2000.00 K, Mean Error = 0.93 % Max Error = 1.72 %

CH_2=CHCH(CH_3)CH=CH_2 + CH_3O_2 = CH_2=CCH(CH_3)CH=CH_2 + CH_3OOH +4.76304030E+02 +3.41386000E+00 +1.98080262E+04 ! Fitting from 500.00 to 2000.00 K, Mean Error = 1.09 % Max Error = 2.13 %

CH_2=CHCH(CH_3)CH=CH_2 + CH_3O_2 = CH_2=CHC(CH_3)CH=CH_2 + CH_3OOH +4.57000000E-03 +4.51110000E+00 +6.89317996E+03 ! Fitting from 500.00 to 2000.00 K, Mean Error = 2.86 % Max Error = 5.74 %

CH_2=CHCH(CH_3)CH=CH_2 + CH_3O_2 = CH_2=CHCH(CH_2)CH=CH_2 + CH_3OOH +2.29081500E+01 +3.76898000E+00 +1.82997307E+04 ! Fitting from 500.00 to 2000.00 K, Mean Error = 1.66 % Max Error = 3.13 %

CH_2=CHCH(CH=CH_2)$_2$ + CH_3O_2 = CH=CHCH(CH=CH_2)$_2$ + CH_3OOH +7.79755158E+03+3.23760000E+00 +2.32794436E+04 ! Fitting from 500.00 to 2000.00 K, Mean Error = 0.77 % Max Error = 1.55 %

CH_2=CHCH(CH=CH_2)$_2$ + CH_3O_2 = CH_2=CCH(CH=CH_2)$_2$ + CH_3OOH +8.40737480E+02 +3.37877000E+00 +2.00737582E+04 ! Fitting from 500.00 to 2000.00 K, Mean Error = 0.99 % Max Error = 1.73 %

CH_2=CHCH(CH=CH_2)$_2$ + CH_3O_2 = CH_2=CHC(CH=CH_2)$_2$ + CH_3OOH +1.17725000E-04 +4.95238000E+00 +5.05441151E+03 ! Fitting from 500.00 to 2000.00 K, Mean Error = 2.91 % Max Error = 5.75 %

! H-atom abstraction reactions of alkynes + CH_3O_2

CH≡CCH_3 + CH_3O_2 = CH≡CCH_2 + CH_3OOH +2.71000000E-03 +4.47903000E+00 +1.31642029E+04 ! Fitting from 500.00 to 2000.00 K, Mean Error = 2.93 % Max Error = 6.12 %

$CH_3C{\equiv}CCH_3 + CH_3O_2 = CH_3C{\equiv}CCH_2 + CH_3OOH$ +8.45000000E-03 +4.51653000E+00 +1.17849585E+04 ! Fitting from 500.00 to 2000.00 K, Mean Error = 2.89 % Max Error = 5.92 %

$CH{\equiv}CCH_2CH_3 + CH_3O_2 = CH{\equiv}CCHCH_3 + CH_3OOH$ +1.67300000E-02 +4.36191000E+00 +1.06015195E+04 ! Fitting from 500.00 to 2000.00 K, Mean Error = 2.77 % Max Error = 5.71 %

$CH{\equiv}CCH_2CH_3 + CH_3O_2 = CH{\equiv}CCH_2CH_2 + CH_3OOH$ +9.05000000E-03 +4.66116000E+00 +1.84330319E+04 ! Fitting from 500.00 to 2000.00 K, Mean Error = 2.89 % Max Error = 5.70 %

$CH{\equiv}CCH(CH_3)_2 + CH_3O_2 = CH{\equiv}CCH(CH_3)CH_2 + CH_3OOH$ +7.03500000E-02+4.02438000E+00 +9.41482103E+03 ! Fitting from 500.00 to 2000.00 K, Mean Error = 2.39 % Max Error = 5.03 %

! H-atom abstraction reactions of ethers + CH_3O_2

$CH_3OCH_3 + CH_3O_2 = CH_3OCH_2 + CH_3OOH$ +1.55750000E-05+5.47428000E+00 +1.29806231E+04 ! Fitting from 500.00 to 2000.00 K, Mean Error = 3.40 % Max Error = 6.72 %

$CH_3OCH_2CH_3 + CH_3O_2 = CH_2OCH_2CH_3 + CH_3OOH$ +6.09570000E-05 +5.22066000E+00 +1.30282990E+04 ! Fitting from 500.00 to 2000.00 K, Mean Error = 3.35 % Max Error = 6.49 %

$CH_3OCH_2CH_3 + CH_3O_2 = CH_3OCHCH_3 + CH_3OOH$ +2.78100000E-02 +4.43126000E+00 +1.16084096E+04 ! Fitting from 500.00 to 2000.00 K, Mean Error = 2.32 % Max Error = 4.51 %

$CH_3OCH_2CH_3 + CH_3O_2 = CH_3OCH_2CH_2 + CH_3OOH$ +7.41750000E-04 +5.02263000E+00 +1.99979265E+04 ! Fitting from 500.00 to 2000.00 K, Mean Error = 3.37 % Max Error = 6.58 %

$CH_3OCH(CH_3)_2 + CH_3O_2 = CH_2OCH(CH_3)_2 + CH_3OOH$ +1.06704000E-05 +5.45363000E+00 +1.27473915E+04 ! Fitting from 500.00 to 2000.00 K, Mean Error = 3.54 % Max Error = 7.12 %

$CH_3OCH(CH_3)_2 + CH_3O_2 = CH_3OC(CH_3)_2 + CH_3OOH$ +1.77329000E-04 +5.07296000E+00 +1.05832470E+04 ! Fitting from 500.00 to 2000.00 K, Mean Error = 3.27 % Max Error = 6.58 %

$CH_3OCH(CH_3)_2 + CH_3O_2 = CH_3OCH(CH_3)CH_2 + CH_3OOH$ +8.38970000E-01 +4.12804000E+00 +1.96952171E+04 ! Fitting from 500.00 to 2000.00 K, Mean Error = 2.15 % Max Error = 4.27 %

! H-atom abstraction reactions of ketones + CH_3O_2

$CH_3C(=O)CH_3 + CH_3O_2 = CH_2C(=O)CH_3 + CH_3OOH$ +8.33539000E-08 +6.01951000E+00 +1.40455709E+04 ! Fitting from 500.00 to 2000.00 K, Mean Error = 3.95 % Max Error = 11.43 %

$CH_3C(=O)CH_2CH_3 + CH_3O_2 = CH_2C(=O)CH_2CH_3 + CH_3OOH$ +7.19008000E-09 +6.20857000E+00 +1.35934349E+04 ! Fitting from 500.00 to 2000.00 K, Mean Error = 3.96 % Max Error = 11.59 %

$CH_3C(=O)CH_2CH_3 + CH_3O_2 = CH_3C(=O)CHCH_3 + CH_3OOH$ +6.08753000E-05 +5.06715000E+00 +1.29590070E+04 ! Fitting from 500.00 to 2000.00 K, Mean Error = 3.51 % Max Error = 7.02 %

$CH_3C(=O)CH2CH3 + CH_3O_2 = CH_3C(=O)CH_2CH_2 + CH_3OOH$ +6.33816000E-12 +7.02659000E+00 +1.48501999E+04 ! Fitting from 500.00 to 2000.00 K, Mean Error = 4.02 % Max Error = 11.74 %

$CH_3C(=O)CH(CH_3)_2 + CH_3O_2 = CH_2C(=O)CH(CH_3)_2 + CH_3OOH$ +4.72277000E-10 +6.55277000E+00 +1.37771906E+04 ! Fitting from 500.00 to 2000.00

K, Mean Error = 4.73 % Max Error = 13.54 %

$CH_3C(=O)CH(CH_3)_2$ + CH_3O_2 = $CH_3C(=O)C(CH_3)_2$ + CH_3OOH +5.83302000E-06 +5.34495000E+00 +1.11584353E+04 ! Fitting from 500.00 to 2000.00 K, Mean Error = 3.69 % Max Error = 7.75 %

$CH_3C(=O)CH(CH_3)_2$ + CH_3O_2 = $CH_3C(=O)CH(CH_3)CH_2$ + CH_3OOH +1.11160000E-01 +4.18651000E+00 +1.81173788E+04 ! Fitting from 500.00 to 2000.00 K, Mean Error = 2.17 % Max Error = 4.30 %
